# Supplementary material for: Attenuated palmitoylation of serotonin receptor 5-HT1A affects receptor function and contributes to depression-like behaviors
Source: Nat Commun. 2019 Sep 2;10:3924. doi: 10.1038/s41467-019-11876-5 (PMC6718429; doi:10.1038/s41467-019-11876-5)
Supplement: Supplementary file 1 — Supplementary Information [file 41467_2019_11876_MOESM1_ESM.pdf]

Supplementary Information

Gorinski et al.

Attenuated palmitoylation of serotonin receptor 5-HT1A in brain affects receptor functions and contributes to depression-like behaviors.

Correspondence to: Ponimaskin.evgeni@mh-hannover.de, Carl-Neuberg-Str. 1, 30625 Hannover, Germany. Phone: +49 511-5324858, Fax: + 49 511-532 8302

Supplementary Figures:

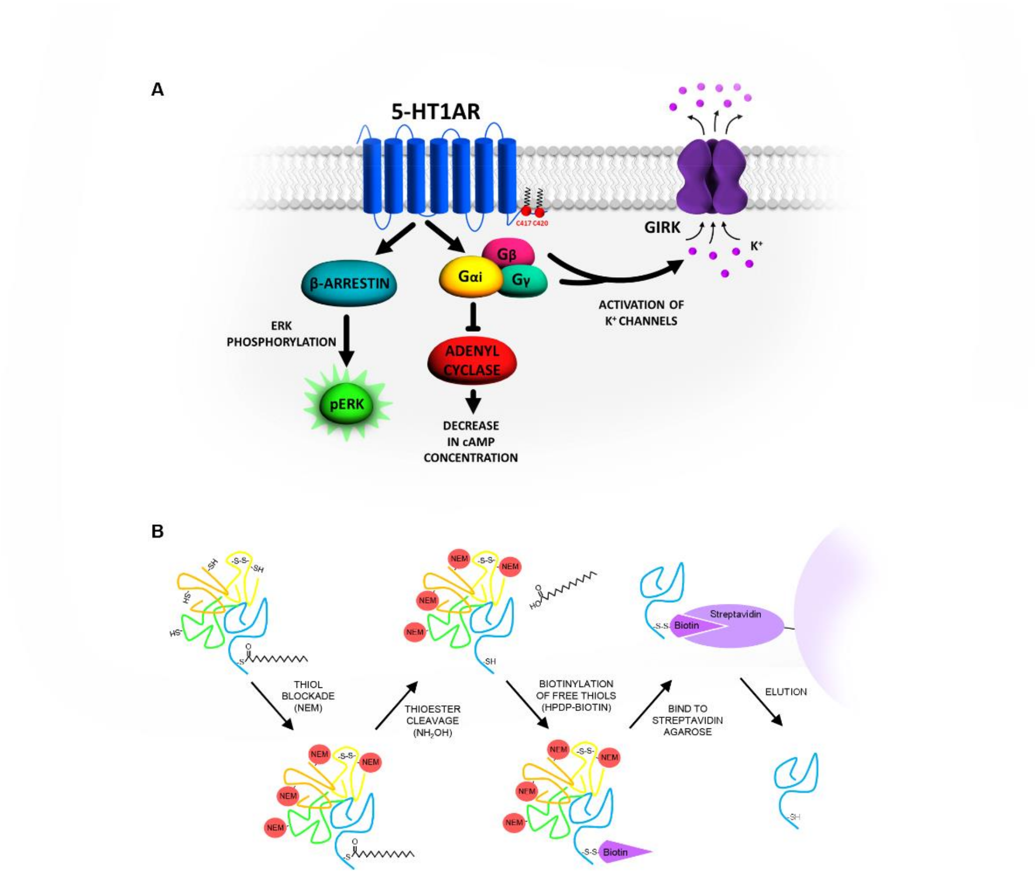

Supplementary Fig. 1, related to Fig. 1. (A) Schematic view of 5-HT1AR structure and receptor-mediated signaling. **(B)** Schematic view of the acyl-biotin exchange (ABE) approach.

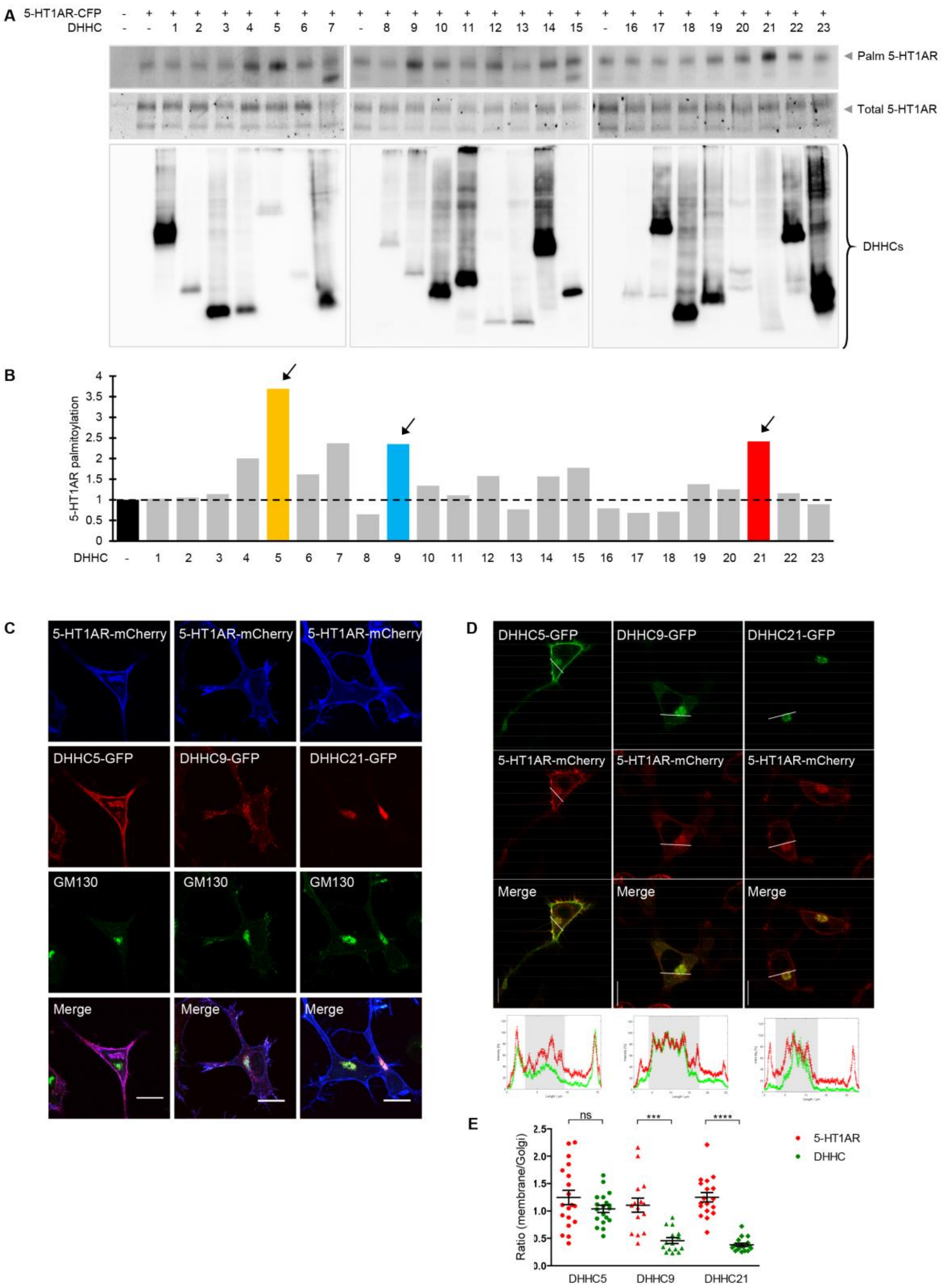

Supplementary Fig. 2, related to Fig. 1. (A) ^{3}H-palmitate incorporation into 5-HT1A-CFP receptor after labeling with [9,10(n)^{3}H] palmitic acid for 2 h at 37°C in neuroblastoma N1E cells co-transfected with receptor and corresponding ZDHHCs. **(B)** Relative changes in 5-HT1AR palmitoylation levels. **(C)** Subcellular distribution of GFP-

tagged ZDHHC5, -9 and -21 and mCherry-tagged 5-HT1AR in N1E cells. Cells were co-transfected with corresponding plasmids, fixed and immunostained with *cis*-Golgi marker (GM130). Scale bar: 20 μm. **(D)** Subcellular distribution of GFP-tagged DHHC5, -9 and -21 and mCherry-tagged 5-HT1AR in living N1E cells. Line scans on the bottom show intensity profiles for 5-HT1AR (red) and ZDHHCs (green). Grey bar shows Golgi compartment. Scale bar: 20 μm. (**E**) Quantification of the intracellular distribution of 5-HT1AR and indicated ZDHHCs. ***, P < 0.001

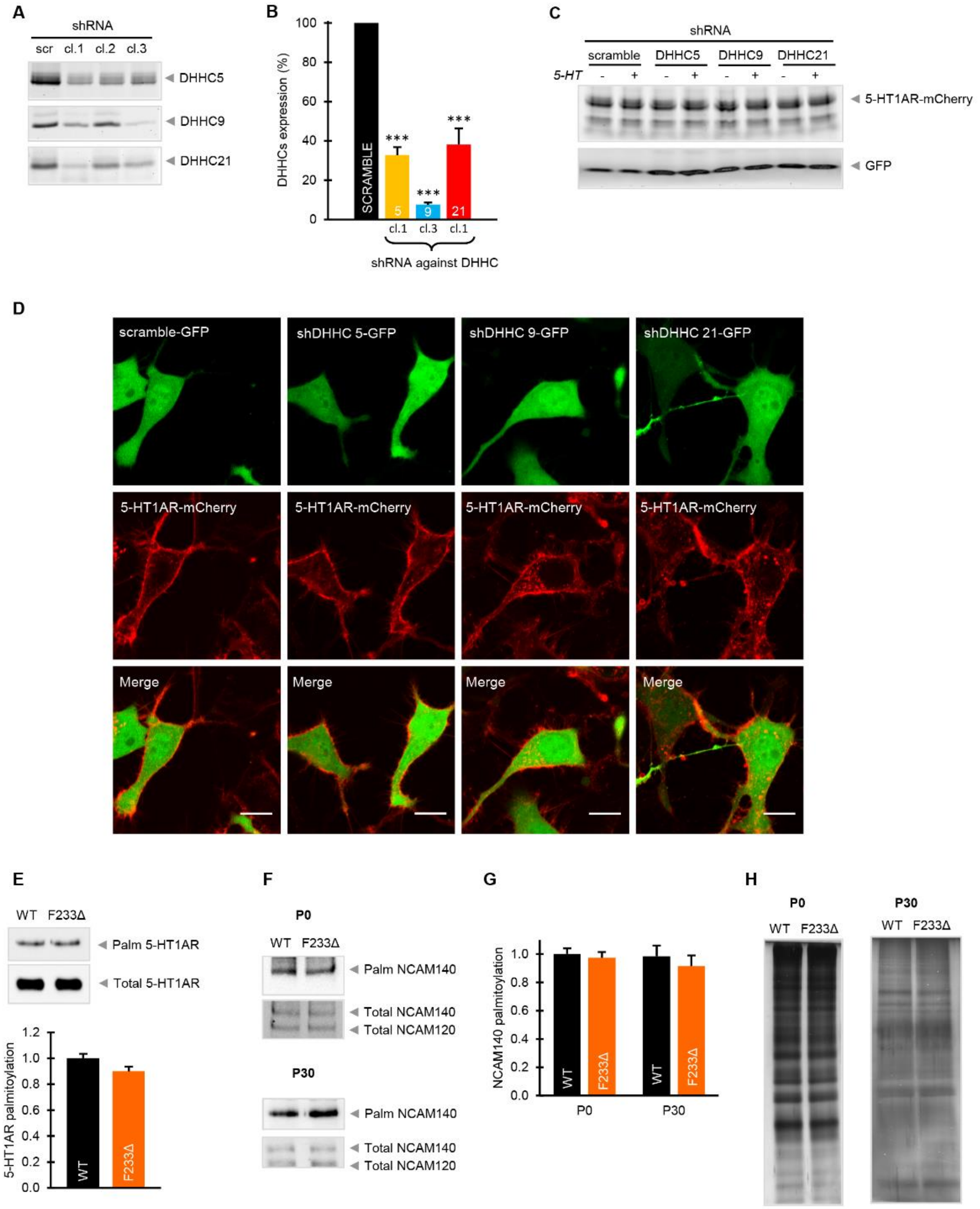

Supplementary Fig. 3, related to Fig. 1. **(A, B)** Knock-down of indicated ZDHHCs with different shRNA clones in N1E cells. Data points represent mean ± SEM from at least three independent experiments (*** P < 0.001). **(C)** Representative western blot showing 5-HT1AR expression in N1E cells after co-transfection together with scramble shRNA or shRNAs against DHHC5, -9 or -21. **(D)** Subcellular distribution of 5-HT1AR-mCherry in living N1E cells after knock-down of endogenous ZDHHC5, -9 or -21 with corresponding shRNAs. Scale bar: 15 µm. **(E)** Brain tissues isolated from the P30 F233Δ Zdhhc21$^{dep/dep}$ and wild-type mice were subjected to the ABE analysis following by quantification (lower panel). **(F)** Palmitoylation of NCAM140 in brain lysates prepared from the newborn (P0) and adult (P30) Zdhhc21$^{+/+}$ (WT) and Zdhhc21$^{dep/dep}$ (F233Δ) mice as assessed by the ABE approach. **(G)** Quantification of the relative NCAM140 palmitoylation in Zdhhc21$^{+/+}$ and Zdhhc21$^{dep/dep}$ mice brains. **(H)**

Representative silver staining demonstrating a global protein palmitoylation in brains from $Zdhhc21^{+/+}$ and $Zdhhc21^{dep/dep}$ mice.

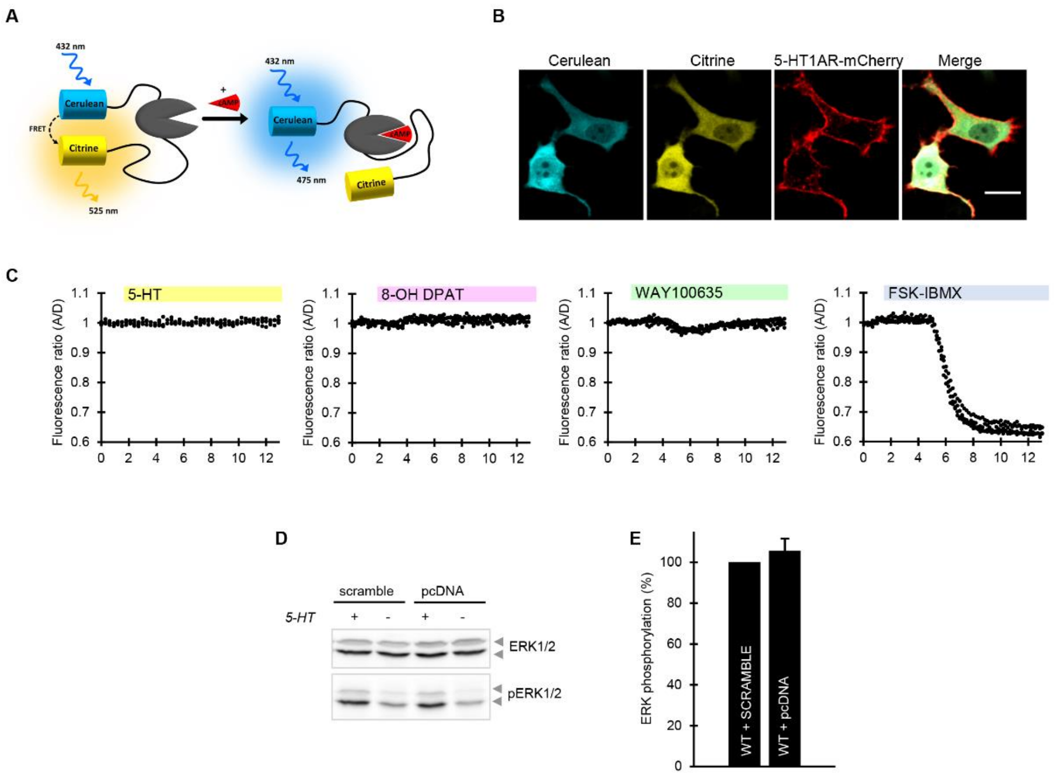

Supplementary Fig. 4, related to Fig. 2. (A) Schematic view of the cAMP FRET-based biosensor CEPAC. N-terminally fused Cerulean is a FRET donor, and N-terminally fused Citrine – FRET acceptor. **(B)** Representative image of living N1E cells co-expressing CEPAC biosensor together with 5-HT1AR-mCherry. Scale bar: 20 μm **(C)** N1E cells were transfected with CEPAC alone and treated either with 5-HT, 8-OH-DPAT (5-HT1AR agonist), WAY100635 (5-HT1AR antagonist), or 1 μM forskolin with 25 μM IBMX. Changes in donor/acceptor ratio were measured in single living cells by confocal laser microscopy. Each trace shows a cAMP response at the single-cell level. **(D)** Representative western blots showing Erk phosphorylation in N1E cells expressing 5-HT1AR and indicated constructs after stimulation with 10 μM 5-HT for 5 min. **(E)** Agonist (i.e., 5-HT) mediated Erk phosphorylation in N1E cells co-expressing 5-HT1AR and indicated constructs is not affected by transfection with scrambled shRNA construct. Data points represent mean ± SEM from at least six independent experiments.

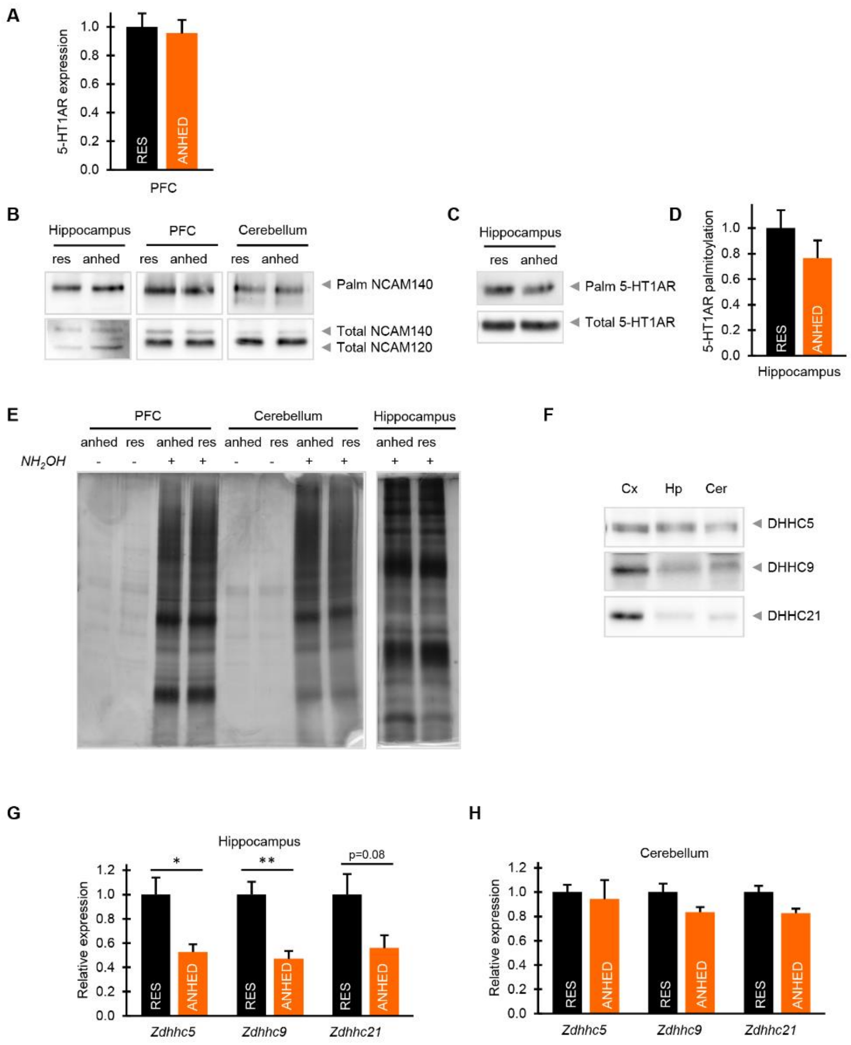

Supplementary Fig. 5, related to Fig. 3. (A) Quantification of the relative 5-HT1AR expression in the PFC from resilient and anhedonic mice. **(B)** Representative western blots showing palmitoylated NCAM140 in hippocampus, PFC and cerebellum from resilient and anhedonic mice as assessed by ABE assay. **(C)** Representative western blots show palmitoylated 5-HT1AR in hippocampus from resilient and anhedonic mice as assessed by ABE assay. (**D**) Quantification of the relative 5-HT1AR palmitoylation in hippocampus from resilient and anhedonic mice. Data points represent mean ± SEM from at least three independent experiments. (**E**) Representative silver staining demonstrating a global protein palmitoylation in the PFC, cerebellum and hippocampus of resilient (res) and anhedonic (anhed) mice. (**F**) Expression levels of ZDHHC5, -9, and -21 in cortex (Cx), hippocampus (Hp) and cerebellum (Cer) of adult C57BL/6J mice. Representative western blots are shown. **(G)** Relative expression levels of Zdhhc5, -9 and -21 in hippocampus and **(H)** cerebellum from resilient (RES) and anhedonic (ANHED) mice. * $P < 0.05$; ** $P < 0.01$.

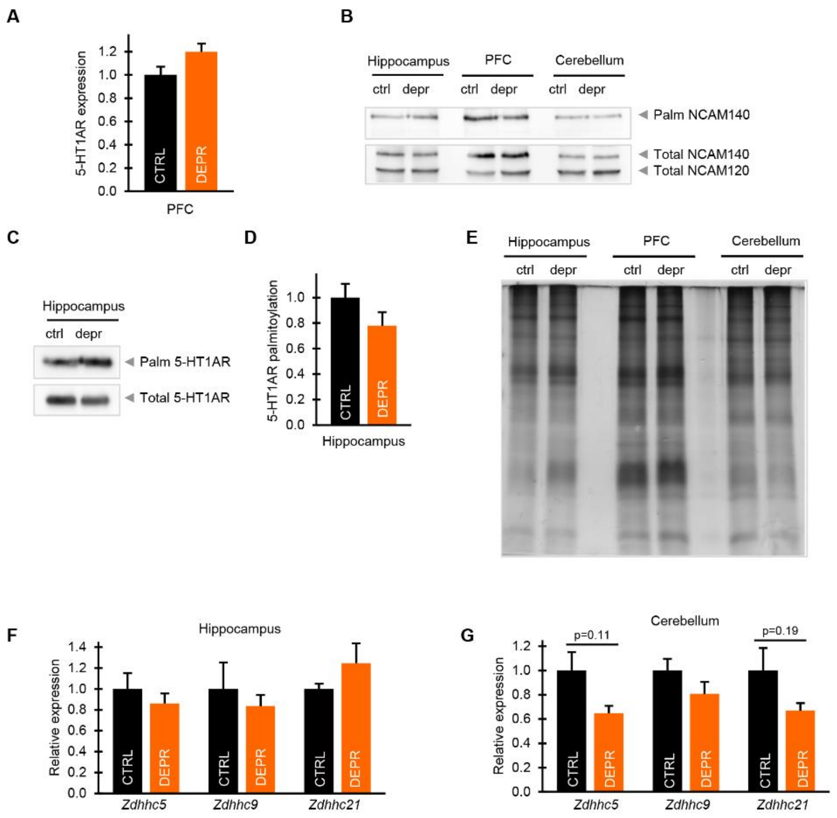

Supplementary Fig. 6, related to Fig. 4. (A) Quantification of the relative 5-HT1AR expression in PFC from rats subjected to restraint stress. **(B)** Representative Western blots after ABE assay show palmitoylated NCAM140 in hippocampus, PFC and cerebellum from rats subjected to restraint stress. **(C)** Representative western blots after ABE assay show palmitoylated 5-HT1AR in hippocampus from rats subjected to restraint stress. **(D)** Quantification of the relative 5-HT1AR palmitoylation in hippocampus from rats subjected to the restraint stress (n =5). **(E)** Representative silver staining demonstrating a global protein palmitoylation patterns in hippocampus, PFC and cerebellum of control animals (ctrl) and rats subjected to restraint stress (depr). **(F)** Relative expression levels of *Zdhhc5, -9 and -21* in hippocampus (n =4) and cerebellum (n = 4) **(G)** from control rats and rats subjected to the restraint stress paradigm. Data points represent mean ± SEM.

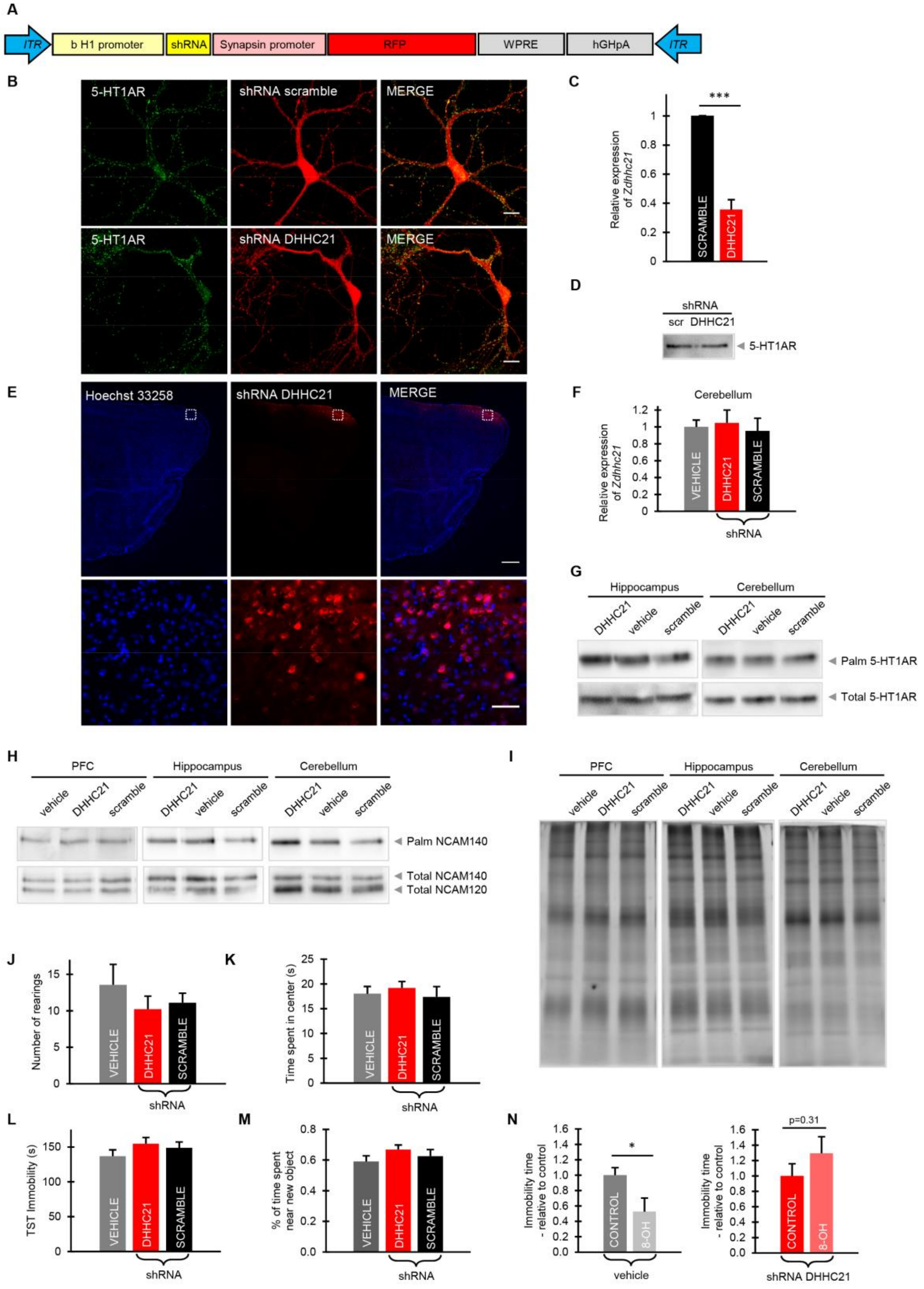

Supplementary Fig. 7, related to Fig. 5.

(A) Schematic view of the adeno-associated viral (AAV) construct encoding for the shRNA against ZDHHC21 together with the red fluorescent protein (RFP). **(B)**

Subcellular distribution of endogenous 5-HT1AR in cultured hippocampal neurons infected with scramble shRNA or shRNA against ZDHHC21. Neurons were infected at DIV6 and fixed for immunostaining at DIV15. Scale bar: 15 µm **(C)** Relative expression levels of *Zdhhc21* in cultured hippocampal neurons infected with scramble shRNA or shRNA against ZDHHC21. Data points represent mean ± SEM from at least four independent experiments (***, $P < 0.001$). **(D)** Representative western blot showing comparable 5-HT1AR levels in cultured hippocampal neurons infected with scramble shRNA or shRNA against ZDHHC21. **(E)** Microscopic images of mouse brain area injected with AAV construct encoding for shRNA against ZDHHC21 and RFP. Adult C57BL/6J mice were infected with AAV constructs bilaterally injected into the PFC. Thirty days after infection brain slices of PFC were stained with DAPI and subjected to confocal microscopy. DAPI and RFP channels are shown. Scale bar: 500 µm (50 µm – zoom area) . **(F)** Relative expression levels of *Zdhhc21* in cerebellum from C57BL/6J mice 30 days after bilateral injection of PFC with AAV constructs encoding for vehicle, scramble shRNA or shRNA against ZDHHC21. **(G)** Representative western blots showing palmitoylation of 5-HT1AR in hippocampus and cerebellum from mice 30 days after injection with indicated AAV constructs. **(H)** Representative western blots showing palmitoylation of NCAM140 in PFC, hippocampus and cerebellum from mice 30 days after injection with indicated AAV constructs. **(I)** Representative silver staining demonstrating a global protein palmitoylation in different brain areas 30 day after injection with indicated AAV constructs. **(J)** Number of rearings in mice treated with vehicle, shRNA against ZDHHC21, or scramble shRNA. **(K)** Percent of time spent in the center of arena for mice treated with vehicle, shRNA against ZDHHC21, or scramble shRNA. **(L)** Immobility time in the tail suspension test of mice treated with vehicle, shRNA against ZDHHC21, or scramble shRNA. **(M)** Percent of time spent near to new object in the novel object recognition test for mice treated with vehicle, shRNA against ZDHHC21, or scramble shRNA. Data points represent mean ± SEM. At least 9 animals per group were analyzed in each test. **(N)** Relative changes of immobility time in the forced swim test (FST) measured in 3-month-old C57BL6/J male control mice or in animals 30 days after administration of AAV encoding for shRNA against ZDHHC21. FST was carried out 20 min after the single injection of the selective 5-HT1AR agonist 8-OH-DPAT (8-OH-DPAT, 1mg/kg, i.p.). Statistical significance between values is noted (* $P < 0.05$, one-way ANOVA), $n \geq 7$ mice.

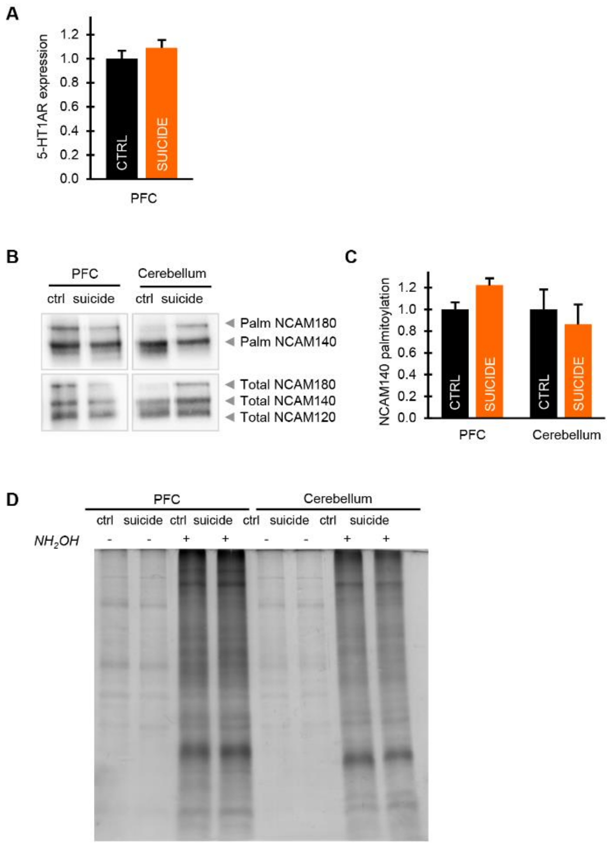

Supplementary Fig. 8, related to Fig. 6. (**A**) Quantification of the relative 5-HT1AR expression in the prefrontal cortex (PFC) samples isolated from control subjects and from individuals with MDD that died by suicide. **(B)** Representative western blots after ABE showing palmitoylated NCAM140 and NCAM180 in the prefrontal cortex (PFC) and cerebellum from control subjects and from individuals with MDD that died by suicide. **(C)** Quantification of the relative NCAM140 palmitoylation in the prefrontal cortex (PFC) and cerebellum from control subjects and from individuals with MDD that died by suicide. **(D)** Representative silver staining demonstrating a global protein palmitoylation in human PFC and cerebellum. Data points represent mean ± SEM.

Supplementary Table 1. *In silico* search for conserved miRNA binding sites in the 3'UTR of *Zdhhc21*

Predicted miRNA	Predicted miRNA binding site (TargetScan)	Predicted miRNA binding site (miRanda)	Predicted miRNA binding site (miRDB)	Predicted miRNA binding site (Pictar)	Conserved miRNA family	Depression association/functional role
miR-338-3p	Yes (7-8mer)	Yes	No	Yes	Broadly conserved	
miR-196b	Yes (8mer)	Yes	No	Yes	Broadly conserved	
miR-200a	Yes (8mer)	Yes	No	Yes	Broadly conserved	
miR-30e	Yes (7-8mer)	Yes	Yes	Yes	Broadly conserved	Depressive disorders [1]
miR-30a	Yes (7-8mer)	Yes	Yes	Yes	Broadly conserved	Neuronal development, depressive disorders [2]

References

1. Xu, Y. *et al.* A polymorphism in the microRNA-30e precursor associated with major depressive disorder risk and P300 waveform. *J. Affect. Disord.* **127,** 332–336 (2010).
2. Mellios, N. *et al.* miR-132, an experience-dependent microRNA, is essential for visual cortex plasticity. *Nat. Neurosci.* **14,** 1240–1242 (2011).
