# Supplementary material for: Attenuated palmitoylation of serotonin receptor 5-HT1A affects receptor function and contributes to depression-like behaviors
Source: Nat Commun. 2019 Sep 2;10:3924. doi: 10.1038/s41467-019-11876-5 (PMC6718429; doi:10.1038/s41467-019-11876-5)
Supplement: Supplementary file 3 — Description of Additional Supplementary Files [file 41467_2019_11876_MOESM3_ESM.pdf]

Description of Additional Supplementary Files

File Name: Supplementary Data 1
Description: Supplementary Table 1A. Proteins identified by mass spectrometry in PFC from mice injected with shDHHC21 and scramble using ABE method. Three independent biological replicated were performed Proteins represented by at least two unique peptides with FDR below 1% are shown.

File Name: Supplementary Data 2
Description: Quantitative analysis based on spectral counts of proteins in PFC from mice injected with shDHHC21 and scramble using ABE method. Three independent biological replicates were performed. Proteins represented by at least two unique peptides with FDR below 1% are shown Mascot score over 25, non-redundant proteins.
