# Supplementary material for: Attenuated palmitoylation of serotonin receptor 5-HT1A affects receptor function and contributes to depression-like behaviors
Source: Nat Commun. 2019 Sep 2;10:3924. doi: 10.1038/s41467-019-11876-5 (PMC6718429; doi:10.1038/s41467-019-11876-5)
Supplement: Supplementary file 6 — Reporting Summary [file 41467_2019_11876_MOESM6_ESM.pdf]

nature research

Corresponding author(s): Evgeni Ponimaskin

Last updated by author(s): Jul 8, 2019

Reporting Summary

Nature Research wishes to improve the reproducibility of the work that we publish. This form provides structure for consistency and transparency in reporting. For further information on Nature Research policies, see Authors & Referees and the Editorial Policy Checklist.

Statistics

For all statistical analyses, confirm that the following items are present in the figure legend, table legend, main text, or Methods section.

n/a	Confirmed	
☐	☒	The exact sample size (*n*) for each experimental group/condition, given as a discrete number and unit of measurement
☐	☒	A statement on whether measurements were taken from distinct samples or whether the same sample was measured repeatedly
☐	☒	The statistical test(s) used AND whether they are one- or two-sided<br>*Only common tests should be described solely by name; describe more complex techniques in the Methods section.*
☒	☐	A description of all covariates tested
☐	☒	A description of any assumptions or corrections, such as tests of normality and adjustment for multiple comparisons
☐	☒	A full description of the statistical parameters including central tendency (e.g. means) or other basic estimates (e.g. regression coefficient) AND variation (e.g. standard deviation) or associated estimates of uncertainty (e.g. confidence intervals)
☒	☐	For null hypothesis testing, the test statistic (e.g. *F*, *t*, *r*) with confidence intervals, effect sizes, degrees of freedom and *P* value noted<br>*Give P values as exact values whenever suitable.*
☒	☐	For Bayesian analysis, information on the choice of priors and Markov chain Monte Carlo settings
☒	☐	For hierarchical and complex designs, identification of the appropriate level for tests and full reporting of outcomes
☒	☐	Estimates of effect sizes (e.g. Cohen's *d*, Pearson's *r*), indicating how they were calculated

Our web collection on statistics for biologists contains articles on many of the points above.

Software and code

Policy information about availability of computer code

Data collection	Data acquisition for electrophysiological experiments was controlled using PATCHMASTER software
Data analysis	- Floating behavior of mice was validated by automated scoring with CleverSys software (CleverSys, VA, US).<br>- Statistical data analysis was performed using Excel 2013 and GraphPad Prism7 Softwares.<br>- ImageJ was used for analysis of fluorescence intensity of images obtained by confocal microscopy<br>- Custom written Matlab scripts were used for preprocessing and analysis of FRET cAMP biosensor data.

For manuscripts utilizing custom algorithms or software that are central to the research but not yet described in published literature, software must be made available to editors/reviewers. We strongly encourage code deposition in a community repository (e.g. GitHub). See the Nature Research guidelines for submitting code & software for further information.

Data

Policy information about availability of data

All manuscripts must include a data availability statement. This statement should provide the following information, where applicable:

- Accession codes, unique identifiers, or web links for publicly available datasets
- A list of figures that have associated raw data
- A description of any restrictions on data availability

The data that support the findings of this study are available from the corresponding author (i.e. EP) upon reasonable request.
According to the Nature communications guidelines, we submitted palmitoylomics raw data to the PRIDE repository (PXD012736).
At the current stage, raw data are only visible for the Reviewers with the following account details:
USERNAME: reviewer55596@ebi.ac.uk
PASSWORD: ZhXXV6IV
In case of acceptance, these data will be available for all readers.

Field-specific reporting

Please select the one below that is the best fit for your research. If you are not sure, read the appropriate sections before making your selection.

☒ Life sciences ☐ Behavioural & social sciences ☐ Ecological, evolutionary & environmental sciences

For a reference copy of the document with all sections, see nature.com/documents/nr-reporting-summary-flat.pdf

Life sciences study design

All studies must disclose on these points even when the disclosure is negative.

Sample size	No predetermine sample-size calculation was performed; all sample sizes were based on statistically significant difference between experimental groups. Sample size was selected according to previous experience and publications.
Data exclusions	In general, no data were excluded from the analyses.<br>During miRNA analysis from human samples we applied outlier analysis using Grubb's Test (single outlier) with alpha = 0,05.<br>In addition, Western blots with air bubbles affecting quantification were exluded from analysis.
Replication	All experimental findings showed the reproducibility and were replicated through the repeated experiments.
Randomization	In general, all samples were allocated in random way.
Blinding	- The analysis of cAMP FRET biosensor was done by Matlab script automatically and ROI selection was done blindly.<br>- Blinding was not relevant to electrophysiological analysis of potassium channel measurements, where agonist was applied to neuronal cultures (i.e. before-after analysis).<br>- In case of Western blot, blinding was not relevant because the method used is robust and there are no subjective decisions during data acquisition and analysis

Reporting for specific materials, systems and methods

We require information from authors about some types of materials, experimental systems and methods used in many studies. Here, indicate whether each material, system or method listed is relevant to your study. If you are not sure if a list item applies to your research, read the appropriate section before selecting a response.

Materials & experimental systems

n/a	Involved in the study	
☐	☒	Antibodies
☐	☒	Eukaryotic cell lines
☒	☐	Palaeontology
☐	☒	Animals and other organisms
☒	☐	Human research participants
☒	☐	Clinical data

Methods

n/a	Involved in the study	
☒	☐	ChIP-seq
☒	☐	Flow cytometry
☒	☐	MRI-based neuroimaging

Antibodies

Antibodies used

- 5-HT1AR (1:200 western blot (WB), 1:200 immunocytochemistry (ICC), 1 µg/ml immunoprecipitation (IP); Alomone Labs (ASR-021)
- 5HT1A receptor (1:200 WB; Abcam ab64994)
- p44/42 MAPK (Erk1/2) (1:1000 WB; Cell Signaling Technology 9102)
- phospho-p44/42 MAPK (Erk1/2) (Thr202/Tyr204) (1:1000 WB; Cell Signaling Technology 9101)
- GFP (1 µg/ml for IP; GeneTex GTX26556)
- GFP (Horseradish peroxidase (HRP)) 1:1000 WB; GeneTex LS-C50850-500)
- hemagglutinin (HA)-probe (Y-11) Santa Cruz sc-805 (2 µg IP)
- synaptophysin 1 (1:50 ICC; Synaptic Systems 101 002)
- biotin (HRP; 1:500 WB; Sigma A4541), TGN38 (1:100 ICC; Thermo Scientific MA3-063)
- HA-peroxidase, high affinity (3F10) (1:8000 WB; Roche 12 013 819 001)
- Alexa Fluor® 647 mouse anti-GM130 (1:100 ICC; BD Biosciences 558712)
- ZDHHC9 polyclonal antibody (1:200 WB; Thermo Fisher Scientific PA5-26721)
- ZDHHC21 polyclonal antibody (1:200 WB; Thermo Fisher Scientific)
- DHHC5 antibody (1:200 WB; ProSci Incorporated 54-211)
- NCAM antibodies were kind gifts from Dr. Martina Muehlenhoff (Gerardy-Schahn, R. & Eckhardt, M. Hot spots of antigenicity in the neural cell adhesion molecule NCAM. Int. J. Cancer Suppl. J. Int. Cancer Suppl. 8, 38–42 (1994))

Validation	Blocking peptide provided by the manufacturer (#ASR-021, Alomone Labs) was used to validate the 5-HT1AR antibody. All other primary antibodies were confirmed by manufacturer to be specific and were applied in multiple published studies.

Eukaryotic cell lines

Policy information about cell lines

Cell line source(s)	Mouse neuroblastoma N1E-115 cell line was purchased by ATCC (ATCC® CRL-2263™)
Authentication	N1E-115 cell line was authenticated by ATCC
Mycoplasma contamination	N1E-115 cell line was micoplasma negative as verified by micoplasma test performed by Eurofins Company (former GATC Biotech)
Commonly misidentified lines (See ICLAC register)	No misidentified cell lines were used in the study

Animals and other organisms

Policy information about studies involving animals; ARRIVE guidelines recommended for reporting animal research

Laboratory animals	- 3-month-old male C57BL/6J wild type mice were used for mouse model of stress-induced anhedonia or PFC knock-down of DHHC21 followed by ABE analysis, immunofluorescent microscopy and miRNA analysis.<br>- 3-month-old male Wistar rats were used for restrain stress rat model followed by ABE and miRNA analysis.<br>- Pupes (P1) of C57BL/6J wild type mice were used for preparation of primary neuronal cultures<br>- Pupes (P0) and 3-month-old Zdhhc21 dep/dep mice (PLoS Genet. 2009, 5:e1000748) were used for ABE analysis.
Wild animals	This study did not involve wild animals
Field-collected samples	This study did not involve samples collected from the field
Ethics oversight	Human brain samples from individuals with MDD that died by suicide (DS) and non-psychiatric control subjects, hereafter referred to as normal control (NC) subjects were obtained from the Maryland Brain Collection at the Maryland Psychiatric Research Center, Baltimore. All procedures were approved by the University of Maryland and University of Illinois institutional review boards.<br>All procedures performed on animal were according to the guidelines of the European Communities Council Directive for the care and use of laboratory animals and approved by the respective local governmental bodies (permission 0421/000/000/2013 was issued by General Directory of Ethical Committee of the New University of Lisbon, in accordance with Portuguese Law-Decrees DL129/92, DL197/96 and Ordinance Port.131/97 and of the 1st Warsaw Ethical Committee on animal research (permission no. 554/2013).

Note that full information on the approval of the study protocol must also be provided in the manuscript.
