# Supplementary material for: Attenuated palmitoylation of serotonin receptor 5-HT1A affects receptor function and contributes to depression-like behaviors
Source: Nat Commun. 2019 Sep 2;10:3924. doi: 10.1038/s41467-019-11876-5 (PMC6718429; doi:10.1038/s41467-019-11876-5)

Figure 1A

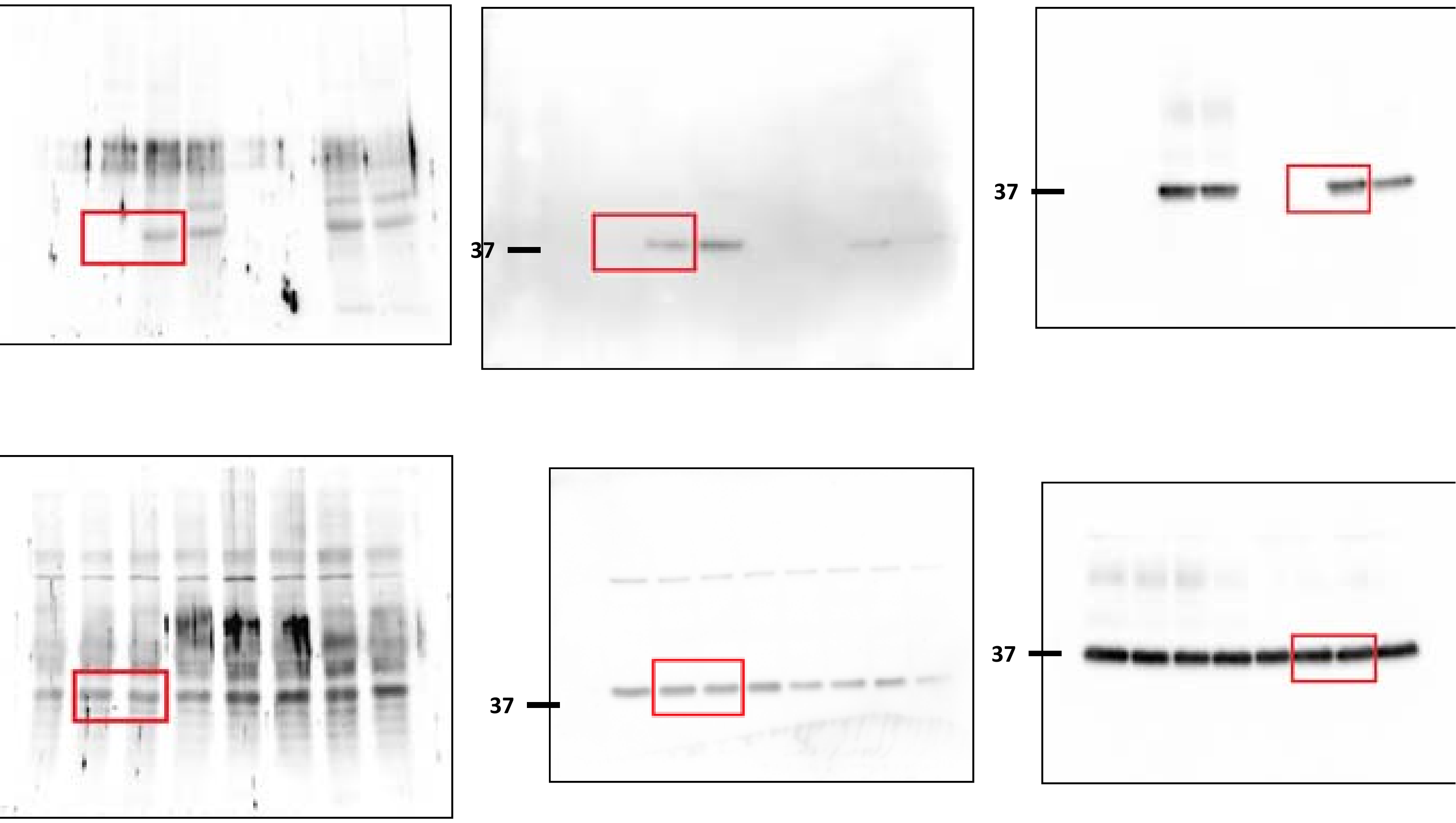

Figure 1B

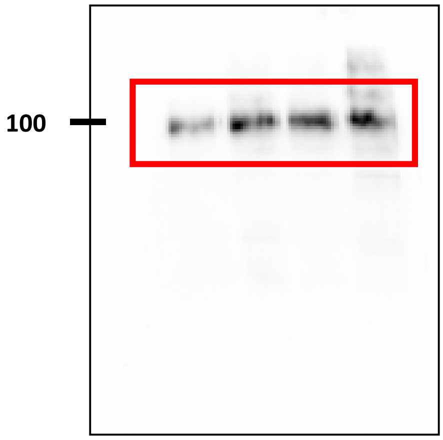

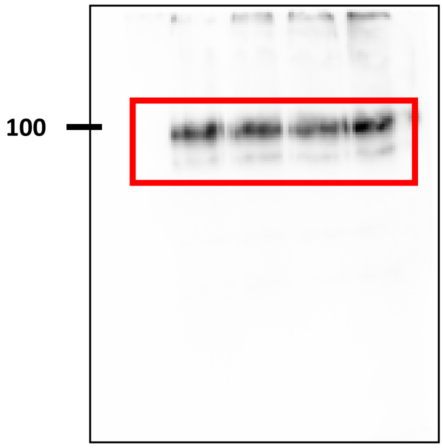

Figure 1D

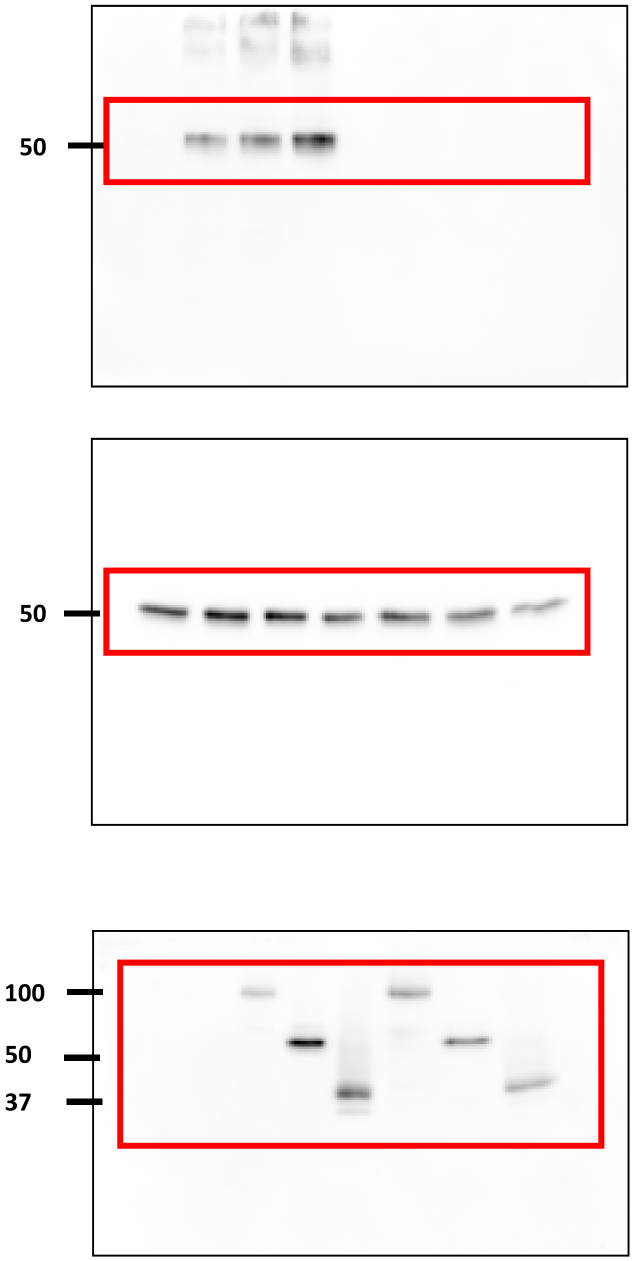

Figure 1E

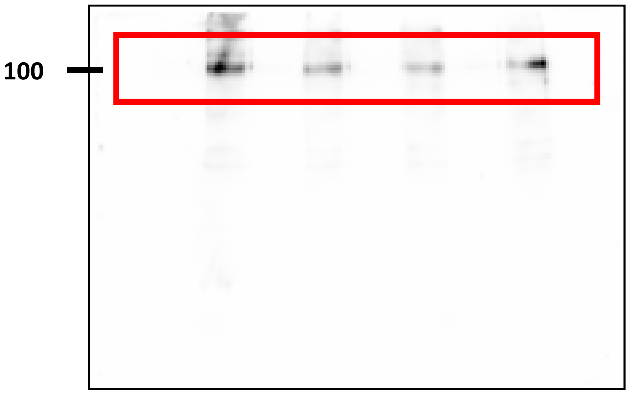

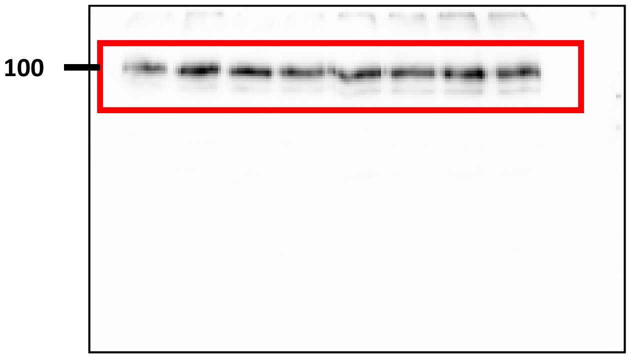

Figure 1G

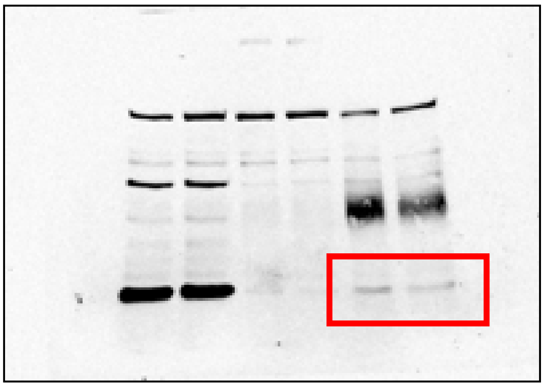

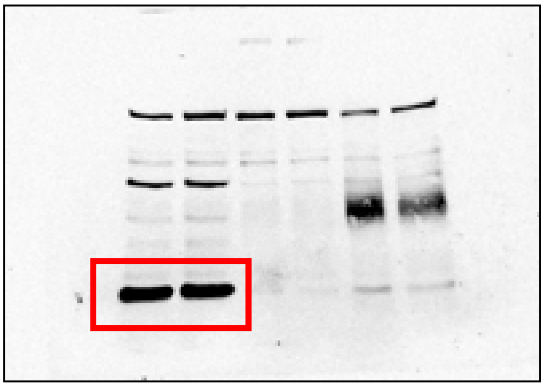

Figure 2D

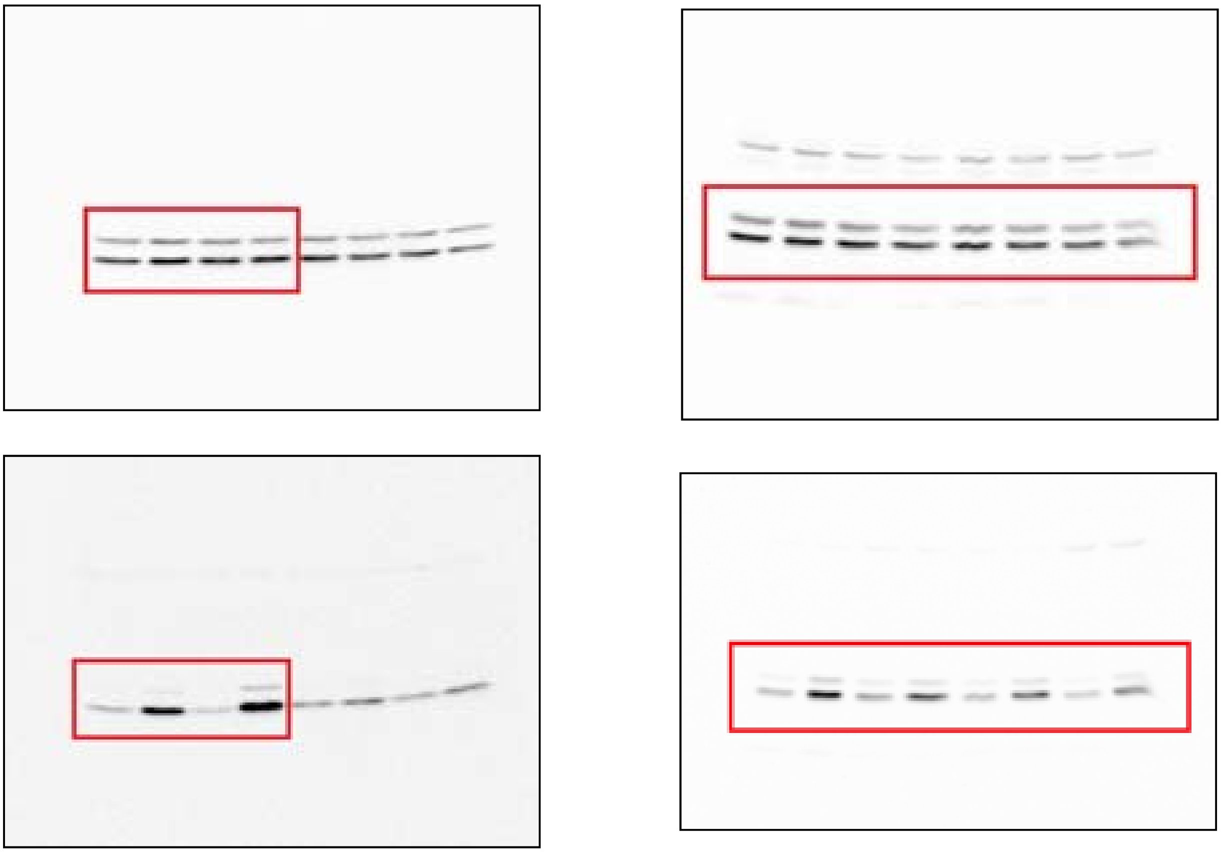

Figure 3D

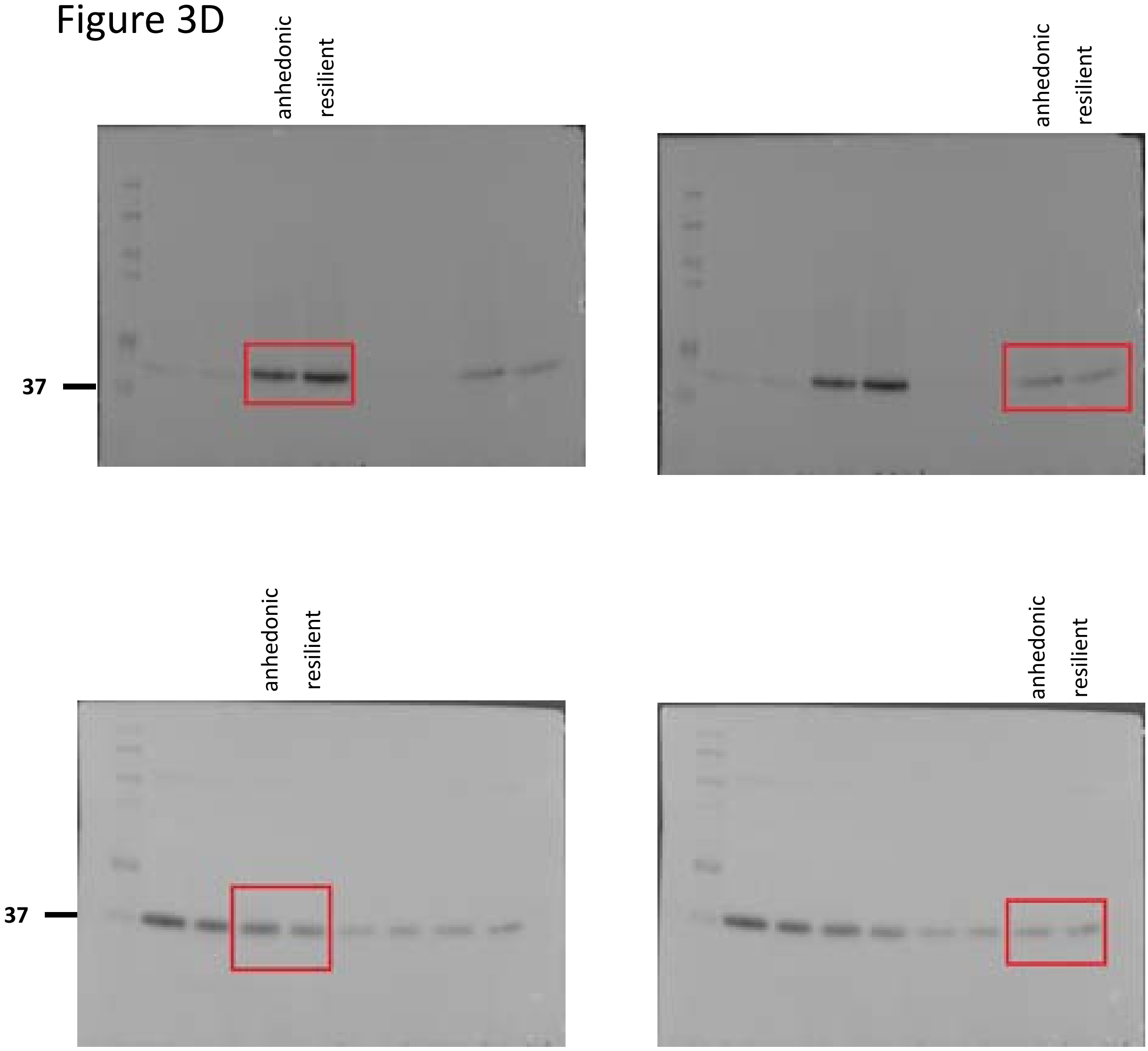
anhedonic
resilient
37
anhedonic
resilient
anhedonic
resilient
37
anhedonic
resilient

Figure 4F

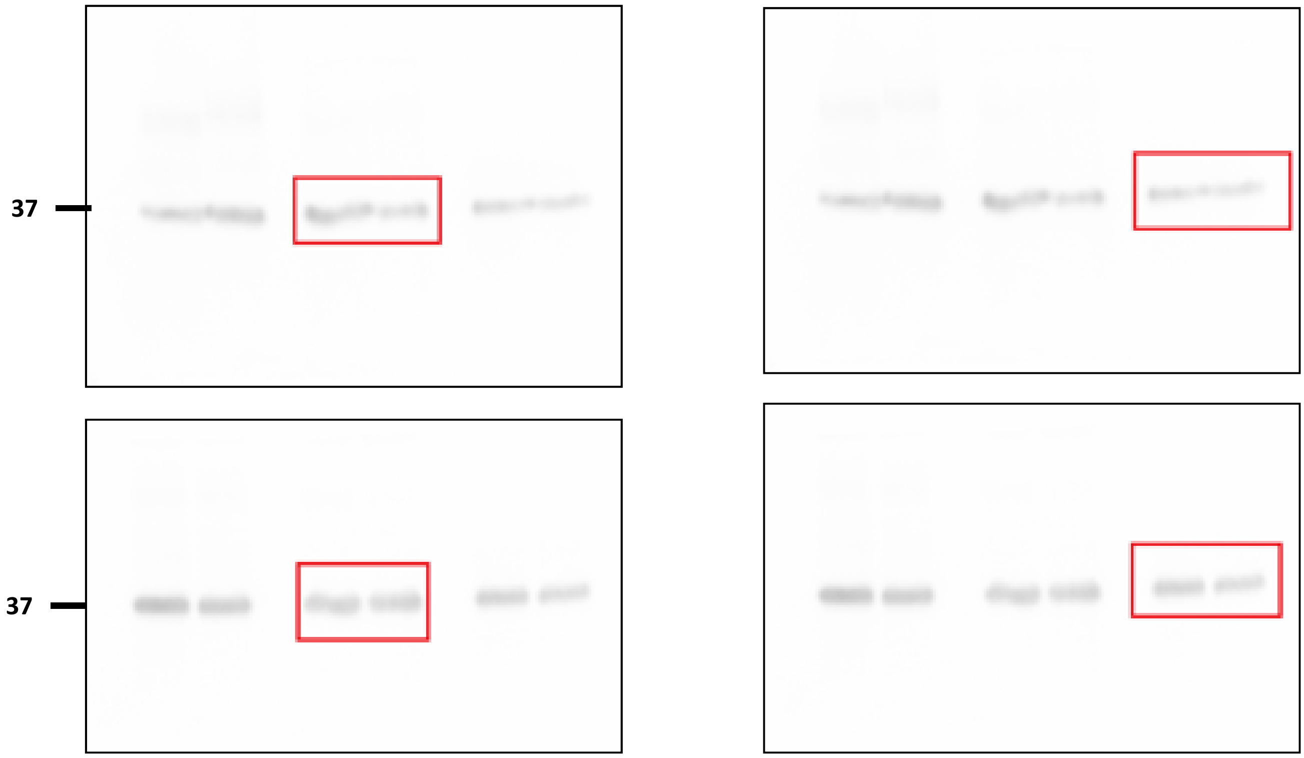

Figure 5C

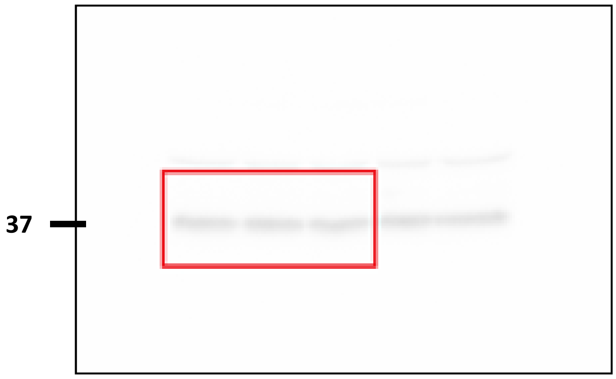

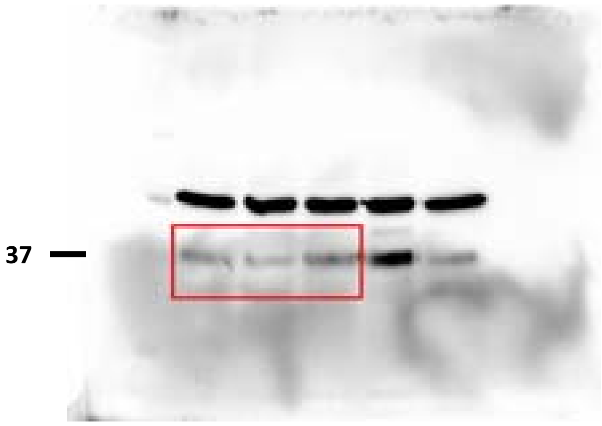

Figure 6A

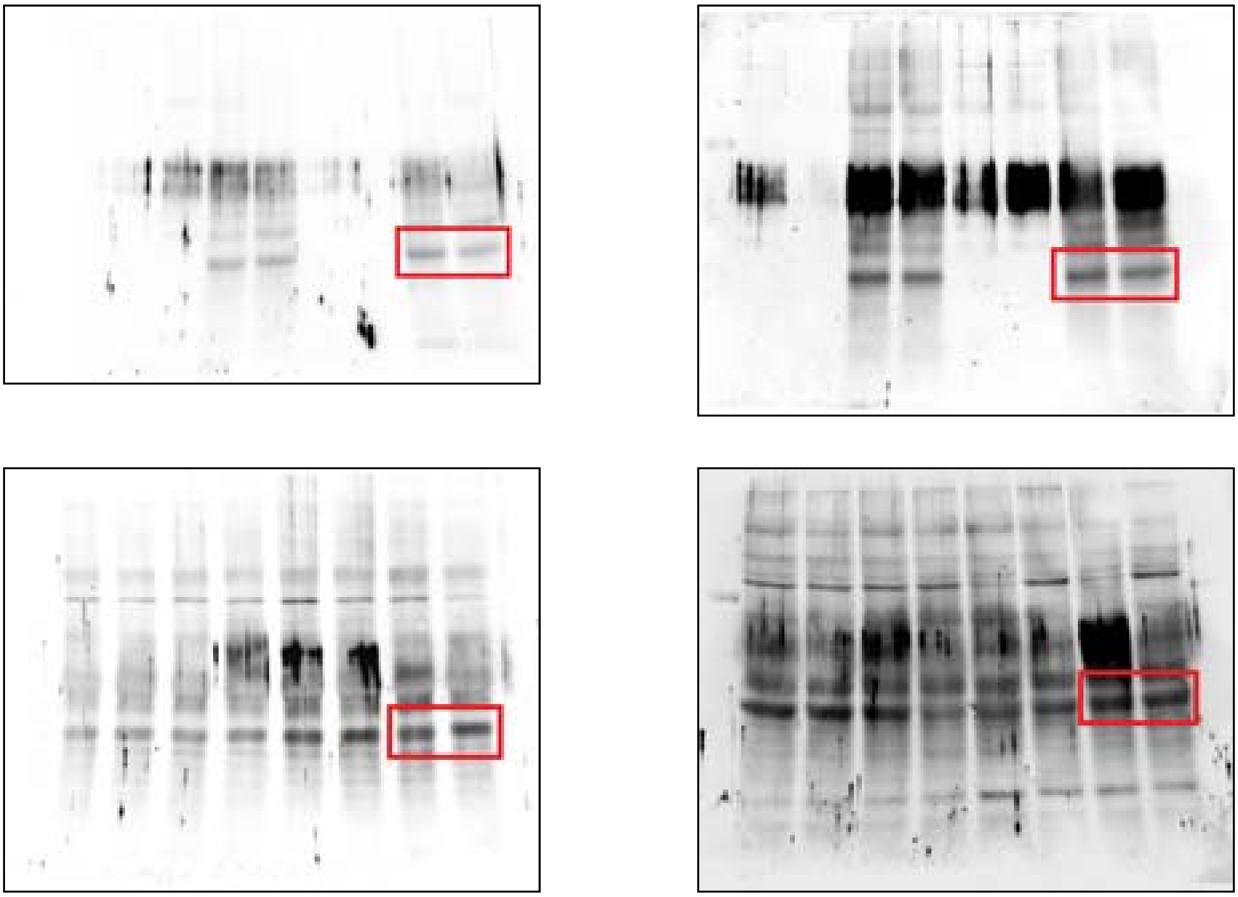

Figure 6E

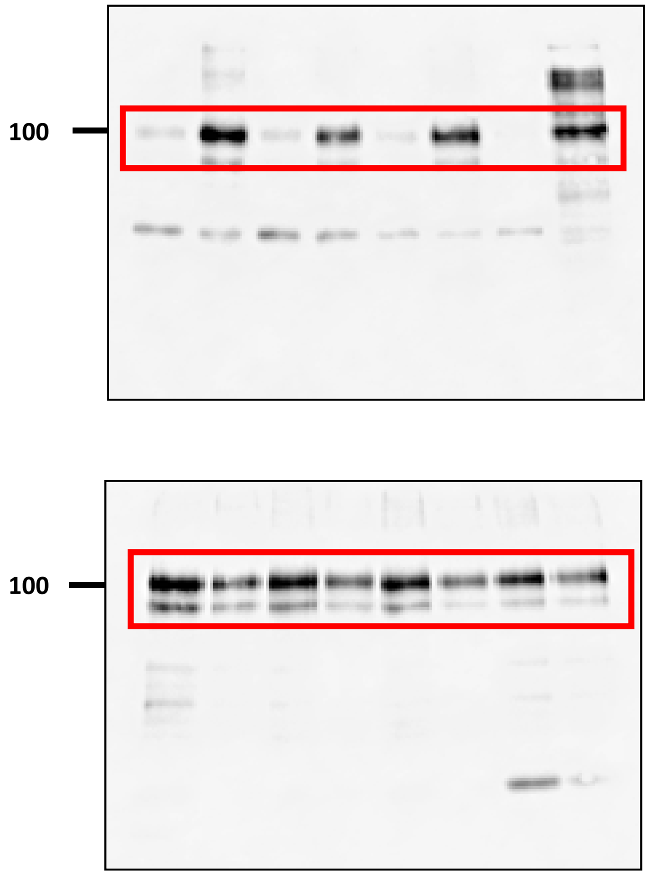

Supplementary Fig. 2A

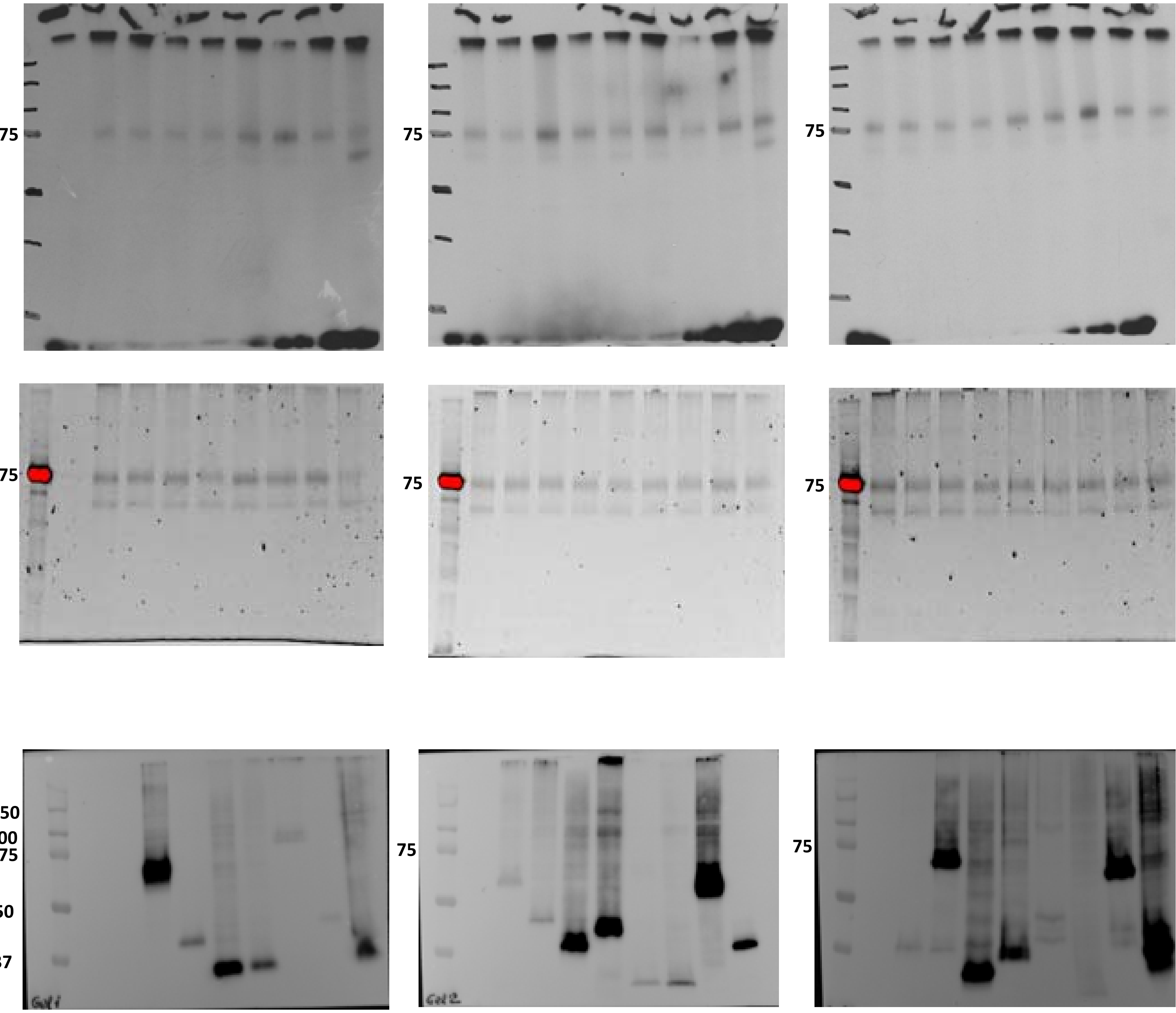

Supplementary Fig. 3A

75
25

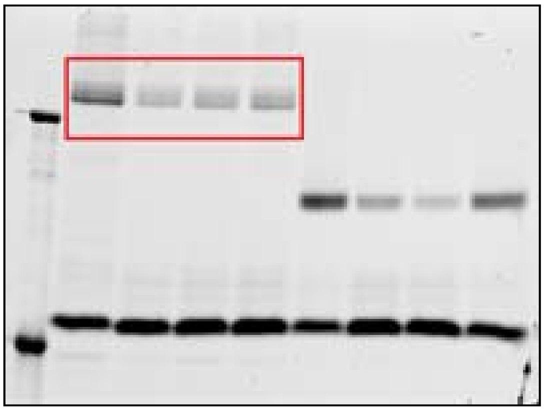

75
25

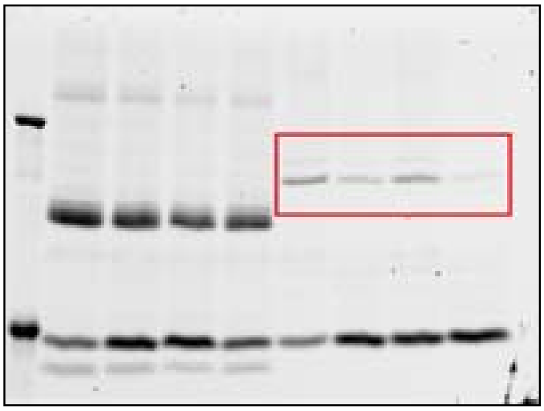

75
25

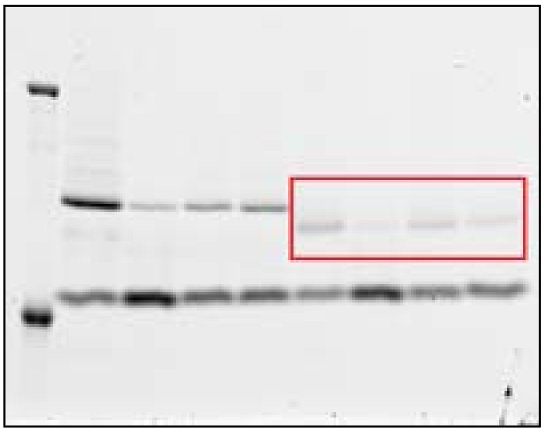

Supplementary Fig. 3C

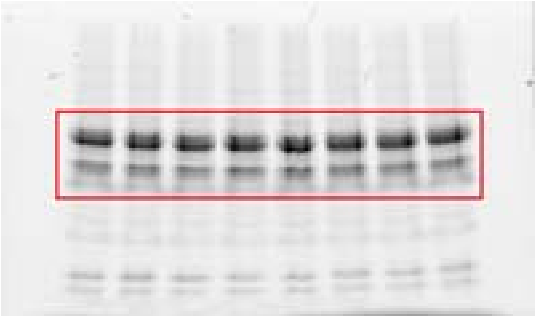

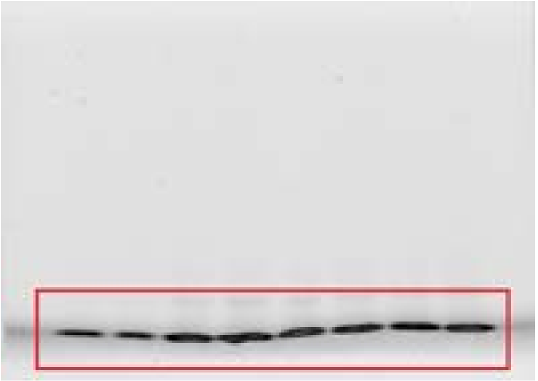

Supplementary Fig. 3E

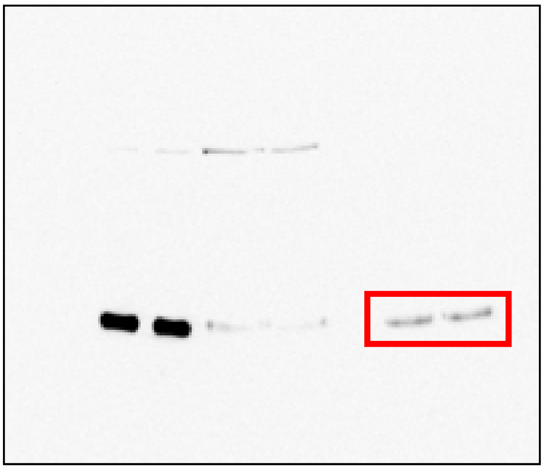

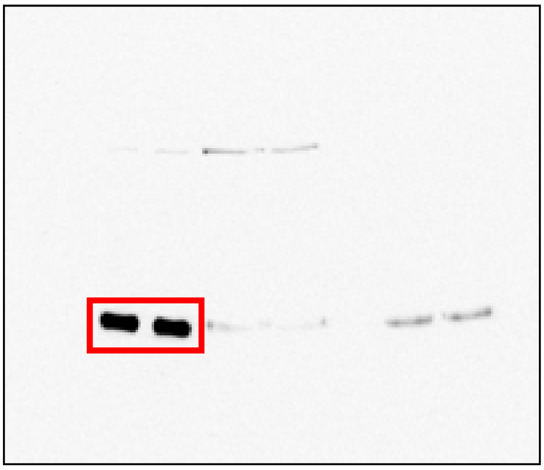

Supplementary Fig. 3F

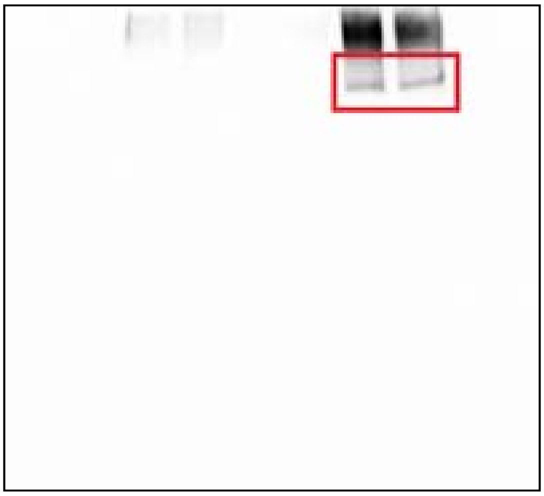

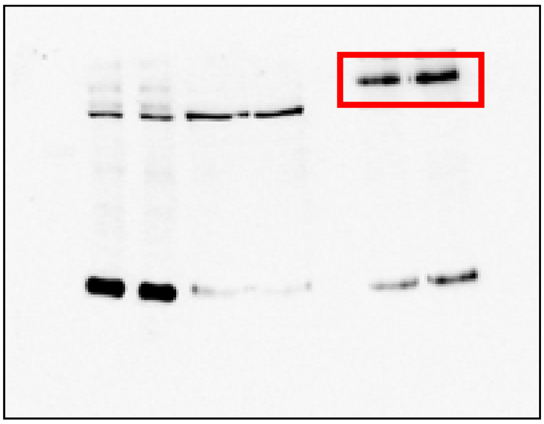

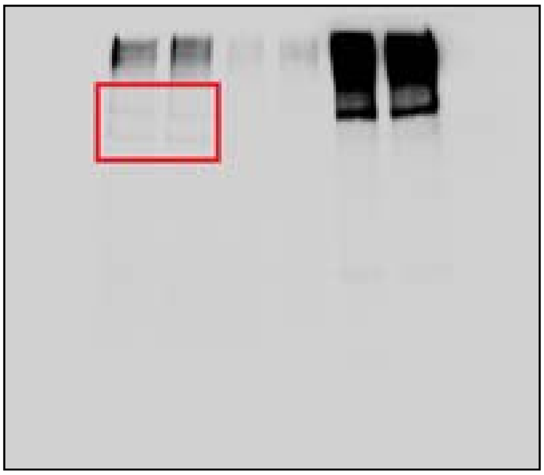

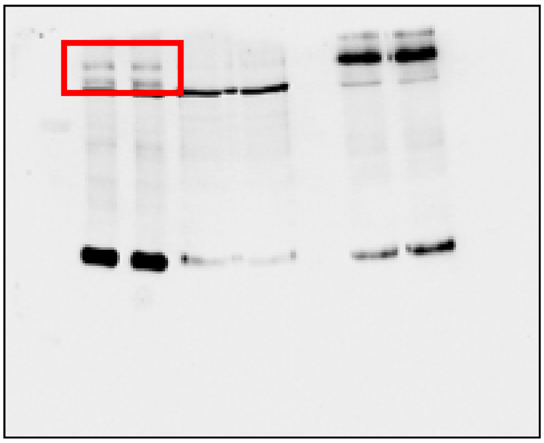

Supplementary Fig. 4D

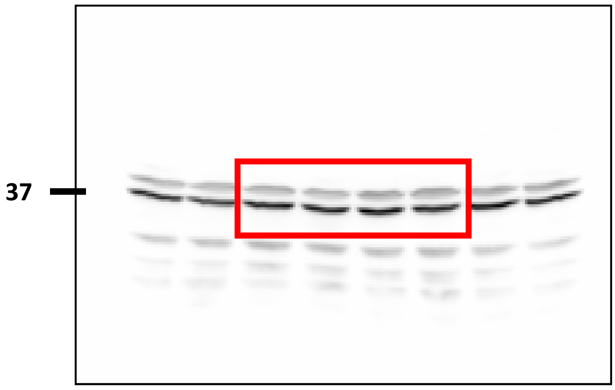

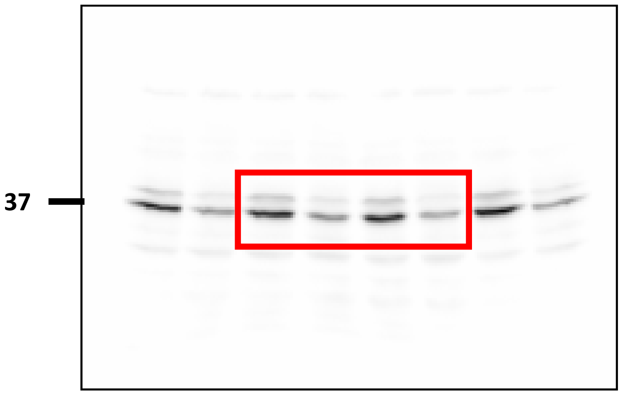

Supplementary Fig. 5B

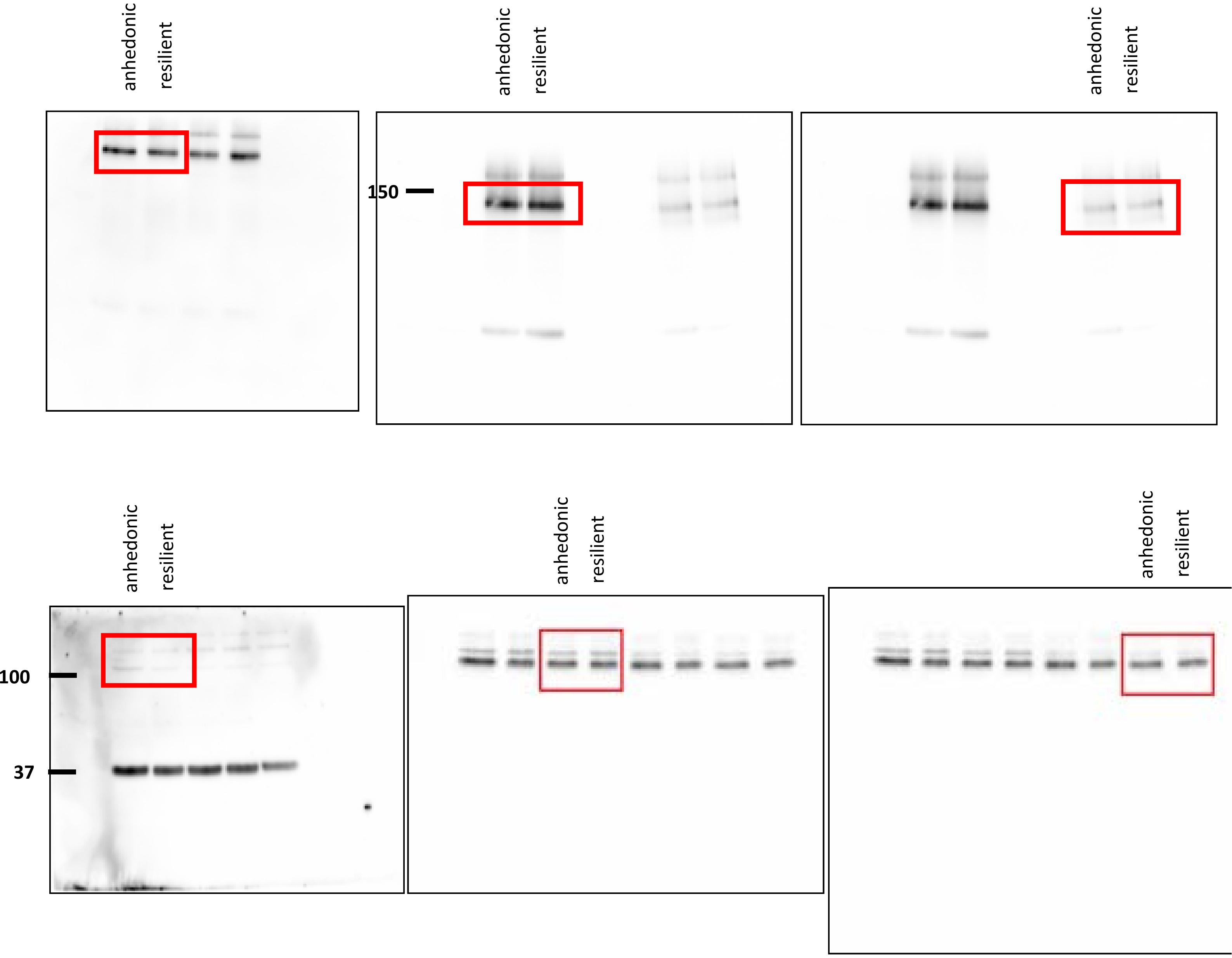

Supplementary Fig. 5C

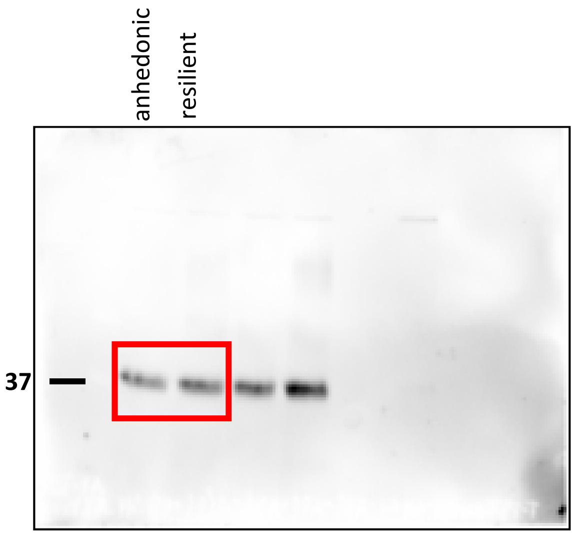

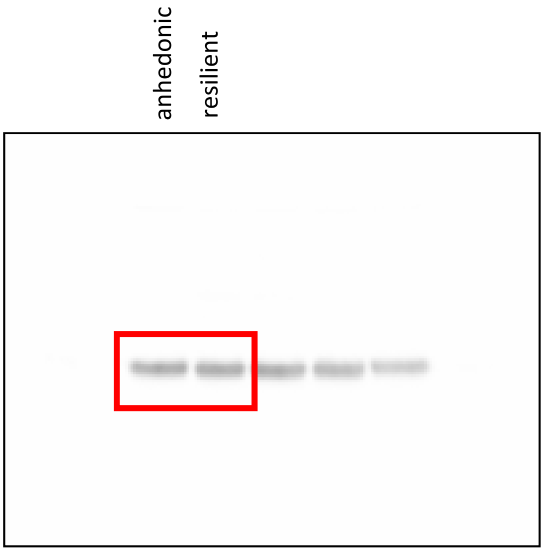

Supplementary Fig. 5F

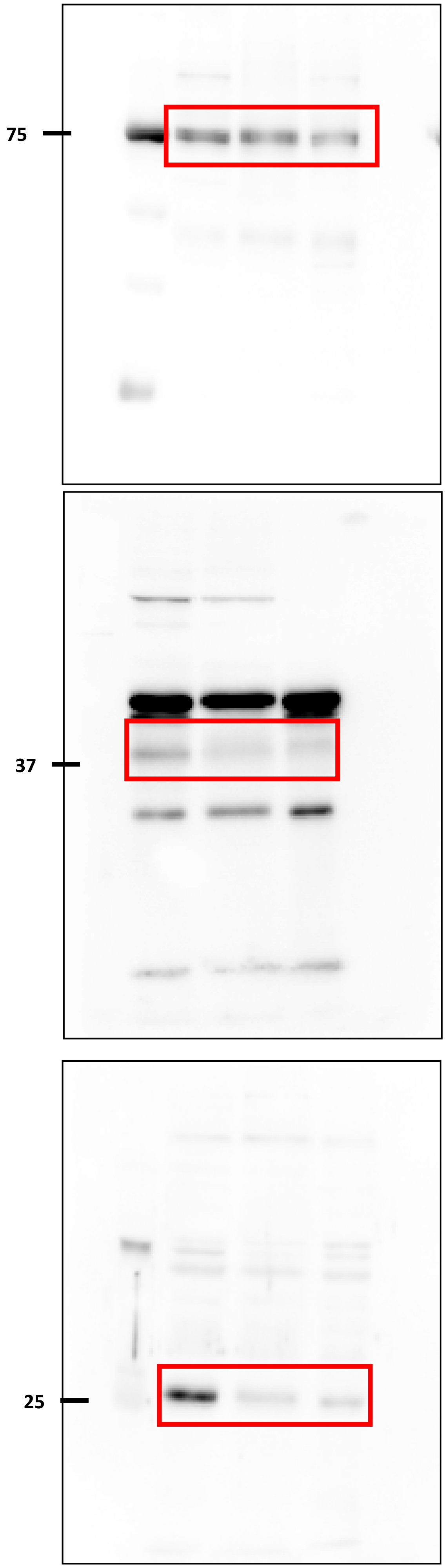

Supplementary Fig. 6B

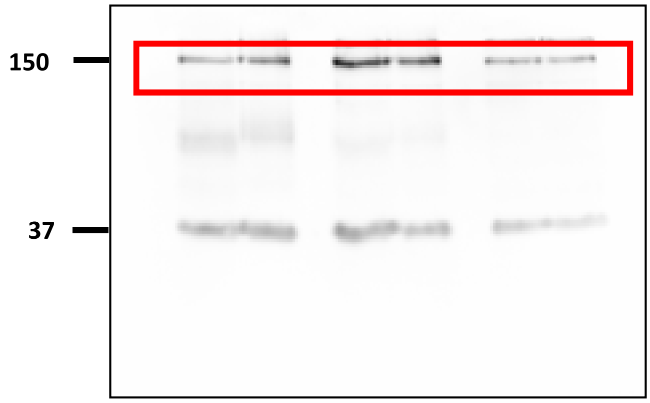

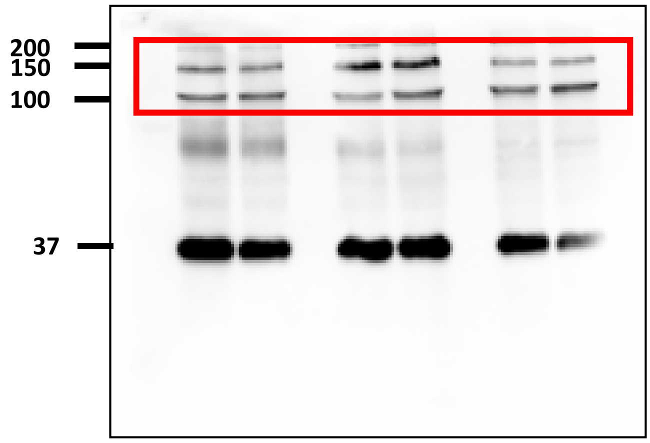

Supplementary Fig. 6C

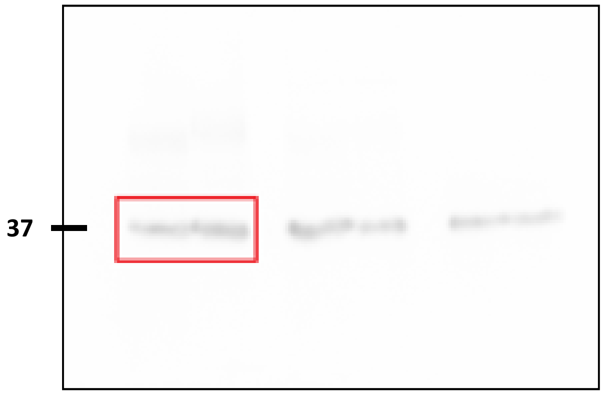

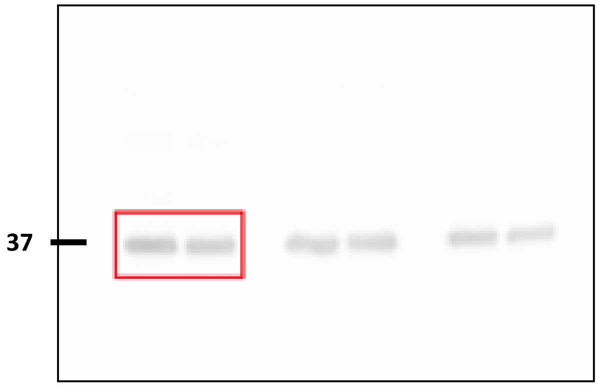

Supplementary Fig. 7D

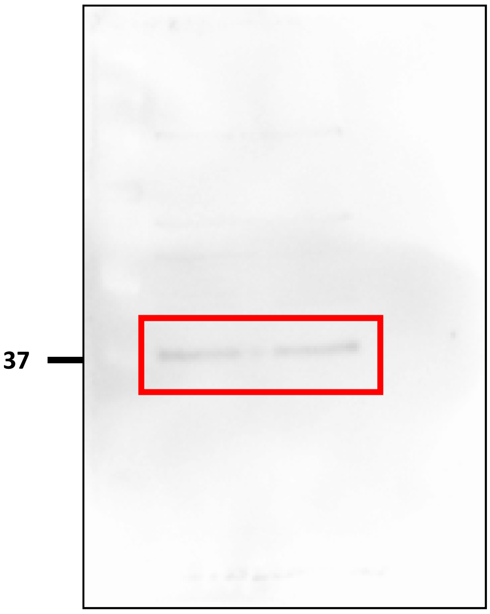

Supplementary Fig. 7G

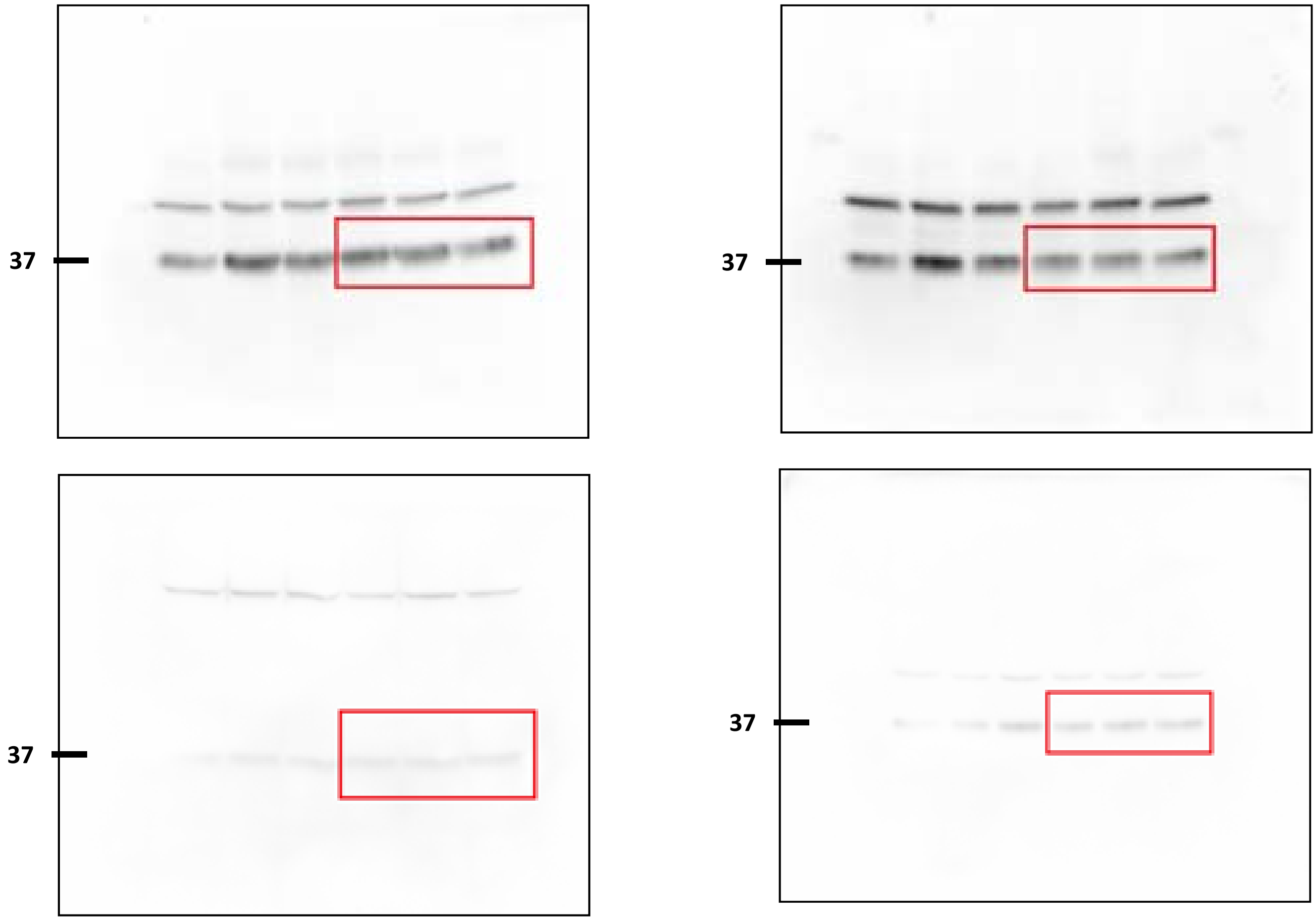

Supplementary Fig. 7H

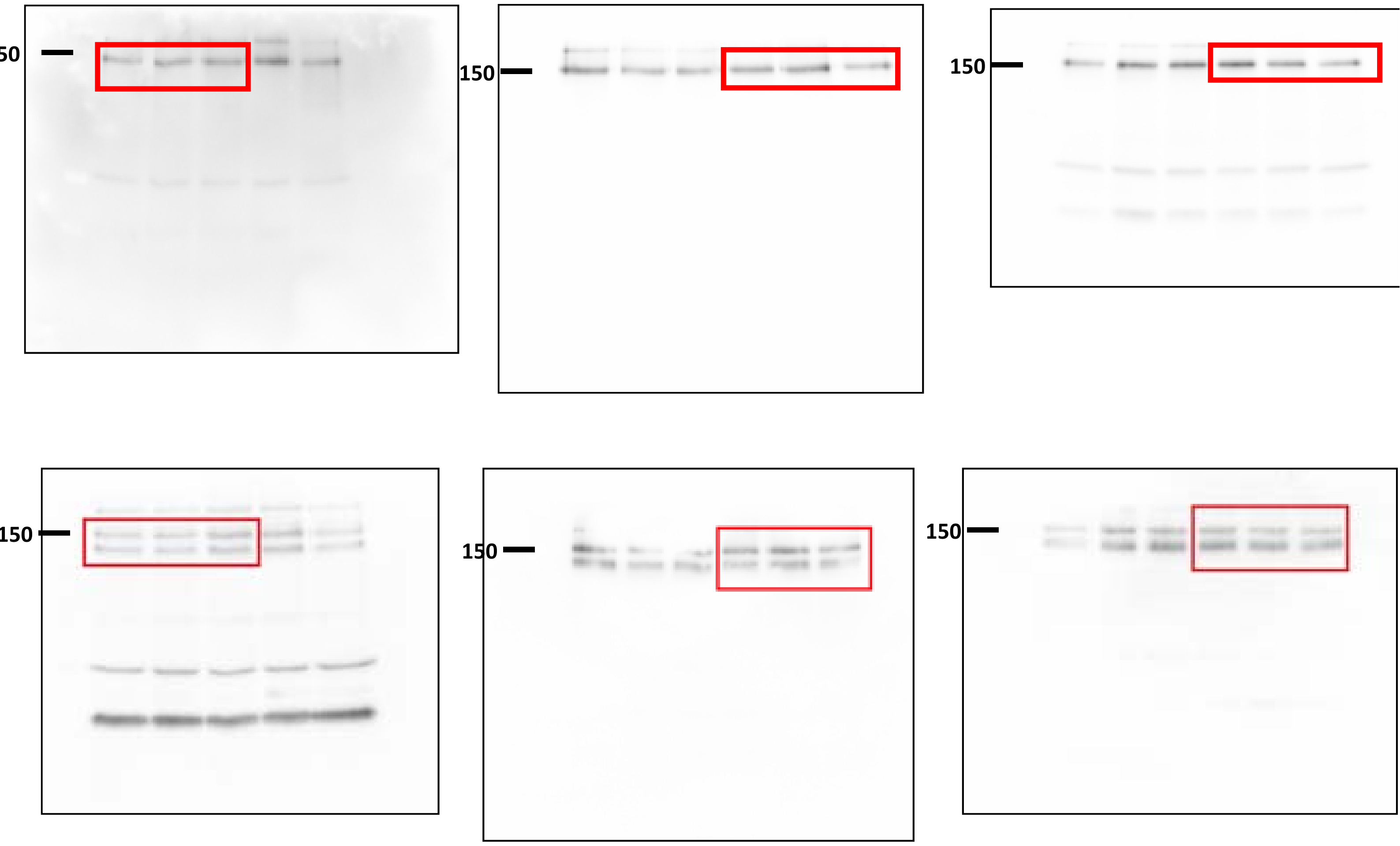

Supplementary Fig. 8B

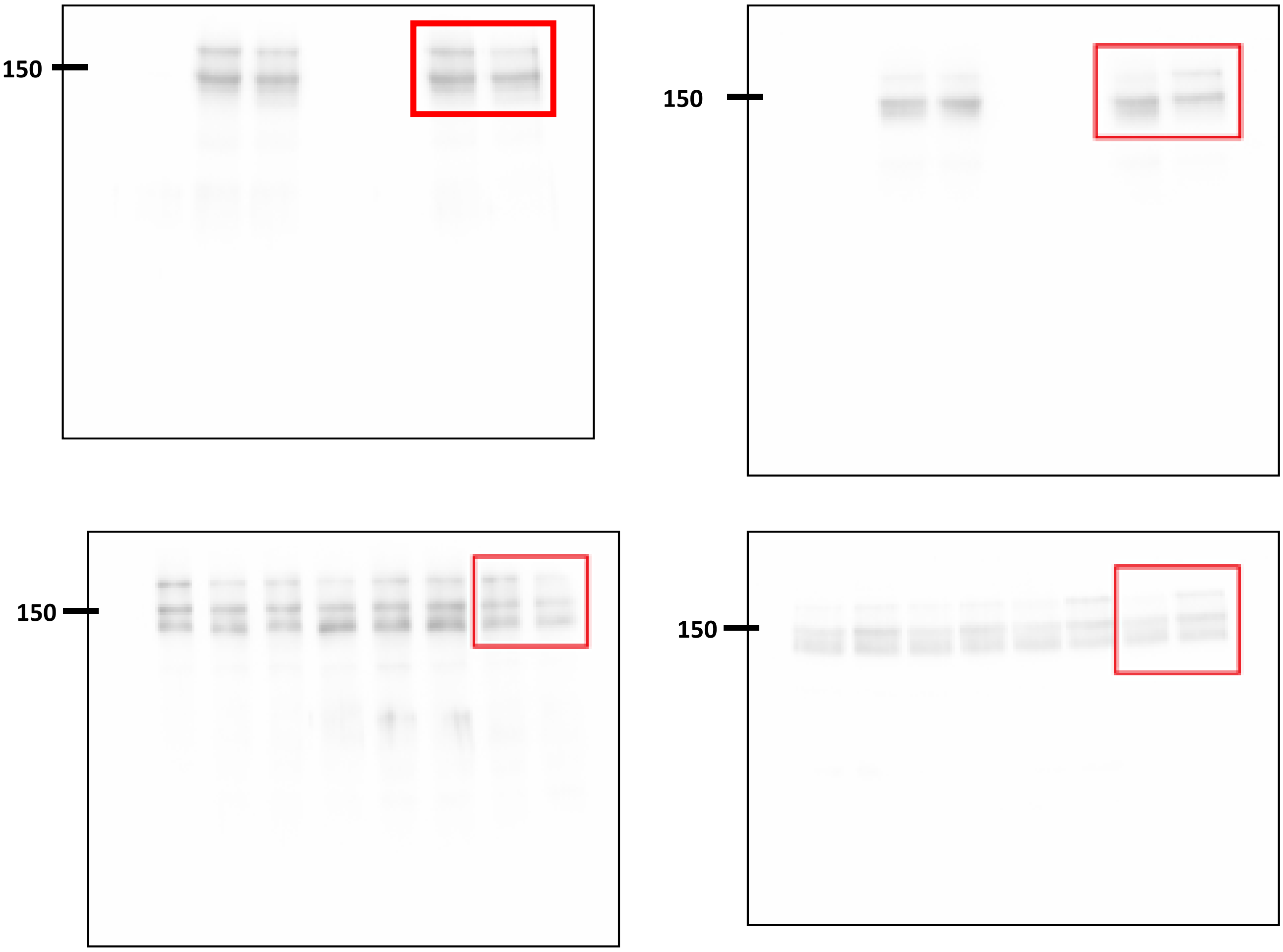

Supplement: Supplementary file 7 — Source Data [file 41467_2019_11876_MOESM7_ESM.zip › Supplementary_data-1.pdf]
